# Supplementary material for: Identifying deleterious noncoding variation through gain and loss of CTCF binding activity
Source: Am J Hum Genet. 2025 Mar 5;112(4):892–902. doi: 10.1016/j.ajhg.2025.02.009 (PMC12081274; doi:10.1016/j.ajhg.2025.02.009)
Supplement: Document S1. Figures S1–S7 and Tables S1 and S2 [file mmc1.pdf]

The American Journal of Human Genetics, Volume *112*

Supplemental information

Identifying deleterious noncoding variation through gain and loss of CTCF binding activity

Colby Tubbs, Mary Lauren Benton, Evonne McArthur, John A. Capra, and Douglas M. Ruderfer

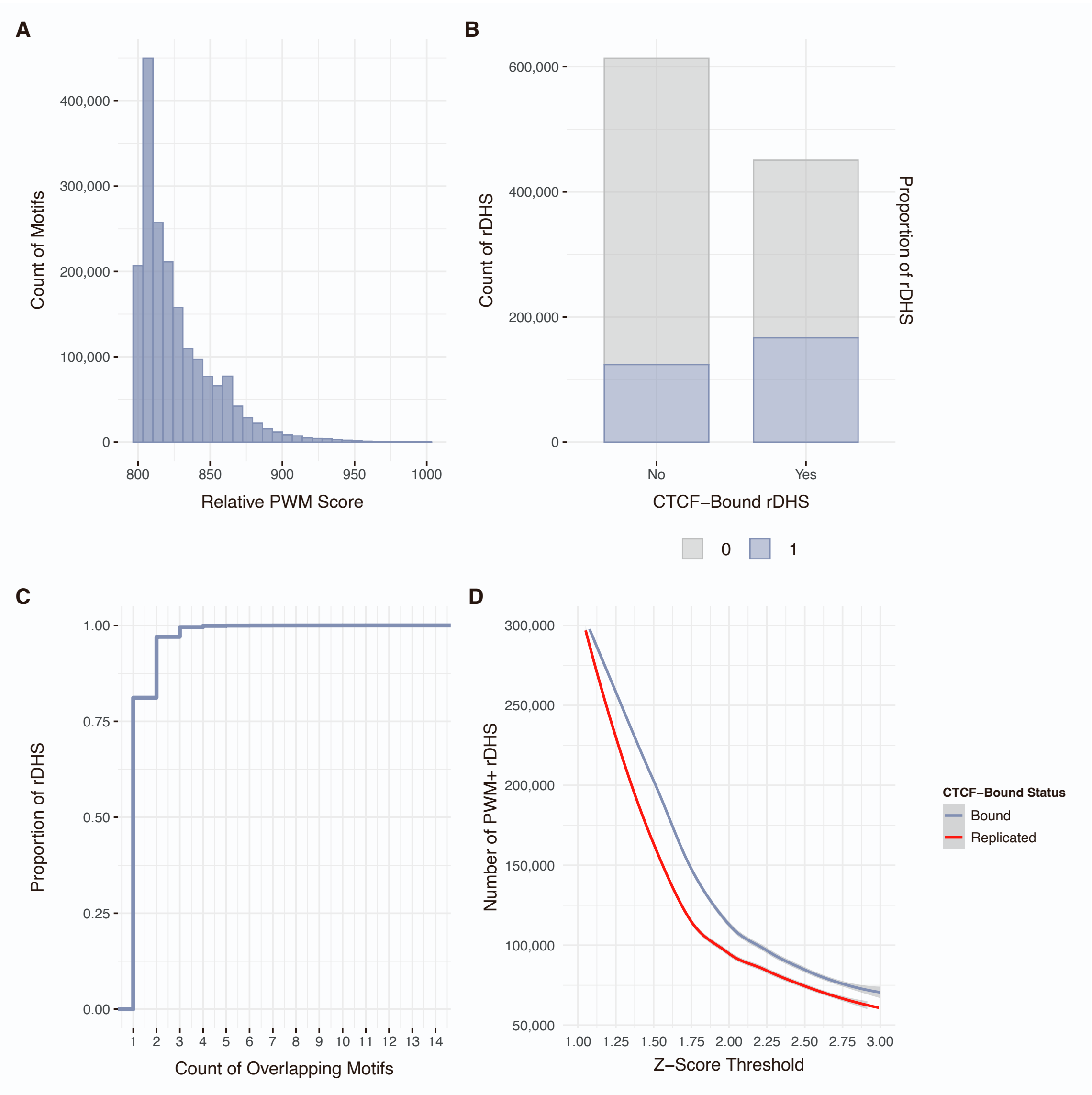

Figure S1. PWMs and ChIP-Seq alone are insufficient for the robust detection of active CTCF binding sequence. A) Distribution of binding energies for all high-quality sequence matches (N = 1,870,772) to the canonical CTCF PWM (JASPAR MA0139.2) in the hg38 reference genome. The threshold for detection was a PWM score of 80% or higher relative to the max possible score for the CTCF PWM. **B)** Counts of rDHS stratified by whether the rDHS is classified as “CTCF Bound” using criteria from ENCODE (Z-score > 1.64 in at least biosample). Each group is colored to reflect the proportion of rDHS that have >=1 overlapping CTCF binding motif. **C)** Cumulative proportion of rDHS by the number of overlapping CTCF motifs after reducing to rDHS with >=1 overlapping motif. **D)** Count of rDHS that would be called as CTCF-bound given the provided Z-score threshold. The “Bound” status requires binding in any of the 214 profiled biosamples. “Replicated” refers to CTCF binding replicated in > 1 biosample. rDHS were subset to those containing at least 1 CTCF PWM match. Plotted are smoothed conditional means using the LOESS method.

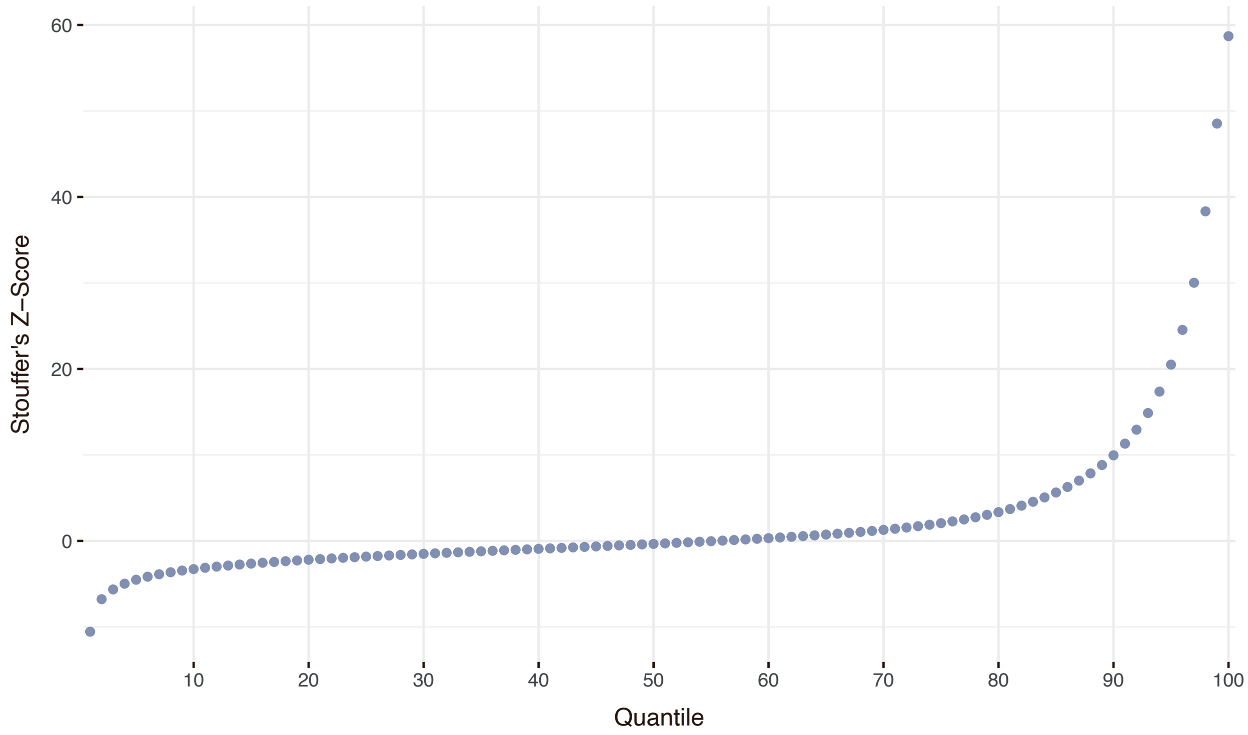

Figure S2. Overview of meta-analyzed Z-scores for each quantile of binding activity. Distributions of meta-analyzed binding activity scores after binning all rDHS into 100 equal sized bins. Each quantile contains approximately 10,638 unique rDHS.

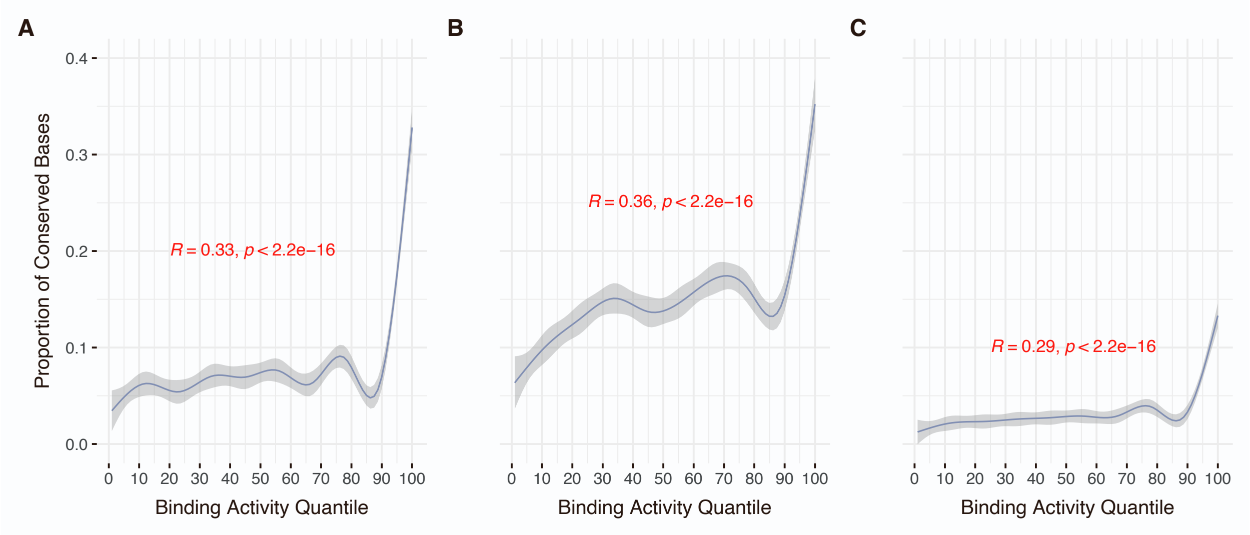

Figure S3. The enrichment of evolutionary sequence conservation at high binding activity quantiles is robust across different conservation metrics. Relationship between evolutionary sequence conservation of CTCF motifs and binding activity quantiles. Conservation was measured as a proportion of conserved CTCF motif positions within each quantile using GERP++, LINSIGHT and Phastcons100 scores. Conservation was measured as a proportion of conserved bases within each bin using a threshold of 2, 0.8 and 0.8 for GERP++, LINSIGHT and Phastcons100 respectively. A-C) Plotted are smoothed conditional means using the LOESS method.

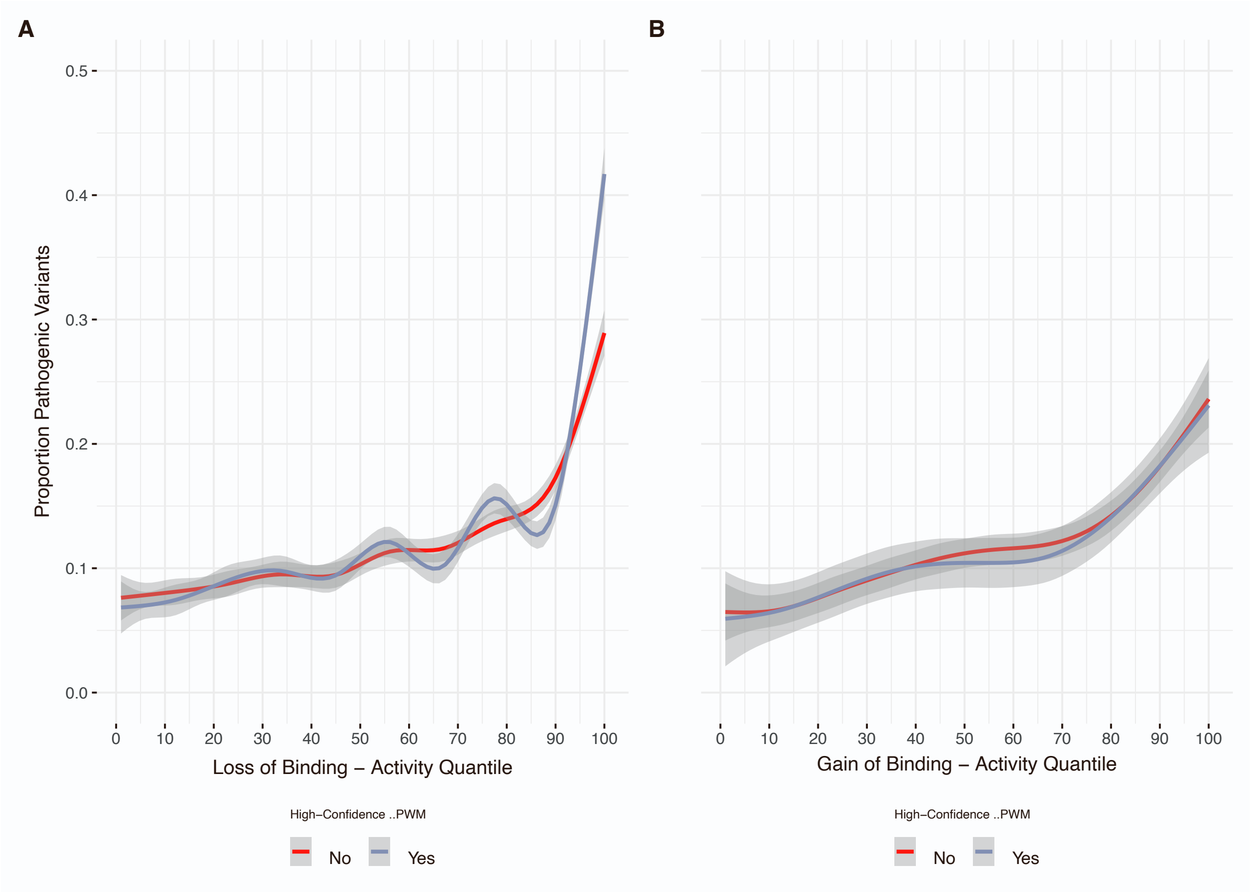

Figure S4. A) Relationship between proportion of putative pathogenic SNVs based on scaled CADD scores and the loss of CTCF binding activity. A scaled CADD score of >=10 was used as a threshold of pathogenic or not. Y-axis displays the proportion of pathogenic SNVs within each activity quantile. Error is plotted as a 95% bootstrapped confidence interval. Colors indicate stratification on the confidence of the ΔPWM call using a threshold of 0.05. **B)** Relationship between the proportion of putative pathogenic SNVs based on scaled CADD scores and the gain of CTCF binding activity assessed using the same approach as in A. A-B) Plotted are smoothed conditional means using the LOESS method.

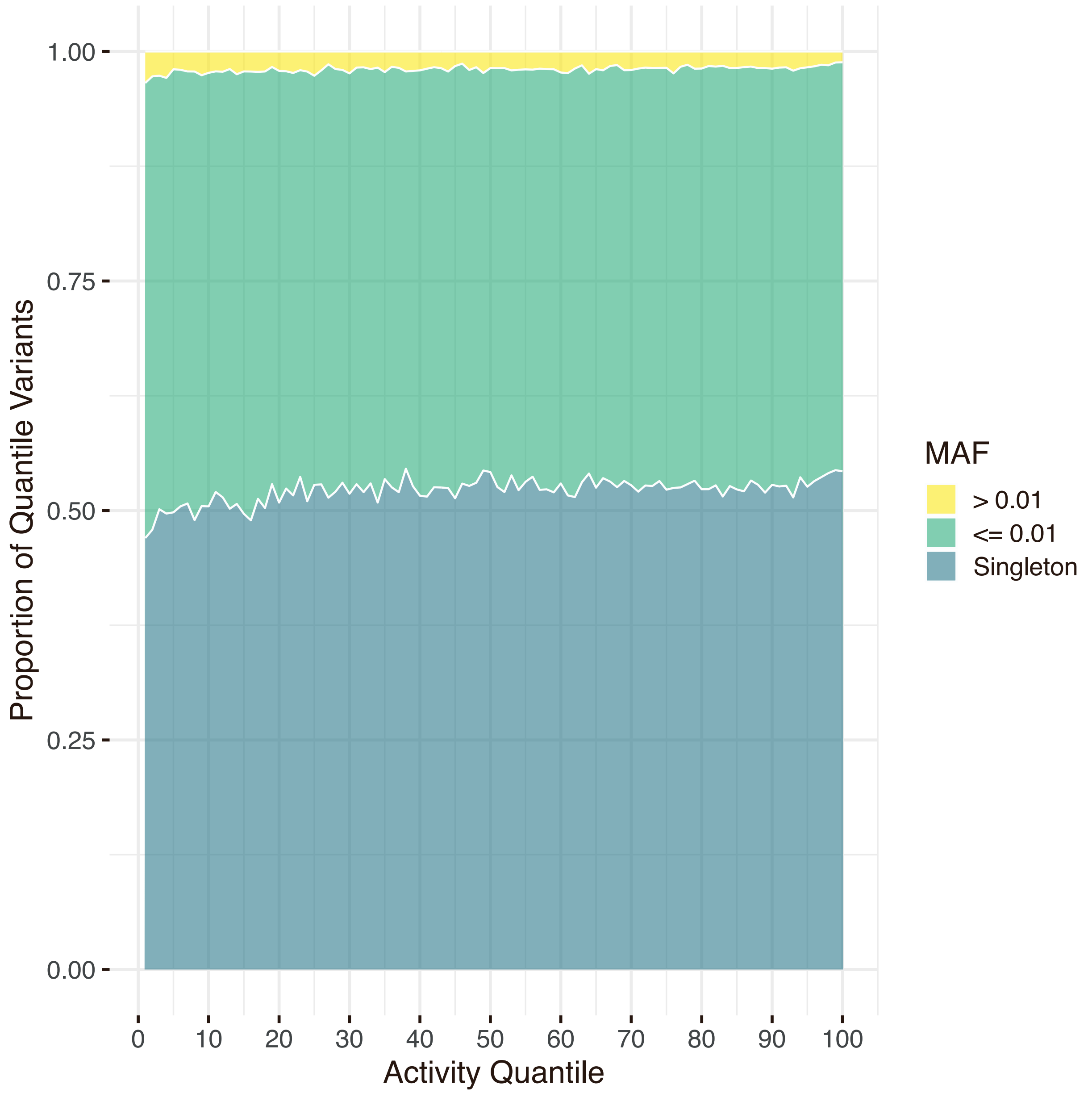

Figure S5. Allele frequency overview of all (n=527,785) significant loss of binding SNVs in gnomAD that disrupt CBSs. These variants represent a subset of all variants identified in the study. Variants were classified as singletons (allele count of 1), MAF <= 0.01, or MAF > 0.01. The X-axis depicts these variants binned by their binding activity quantile. The Y-axis represents the proportion of variants in each quantile belonging to a given allele frequency class.

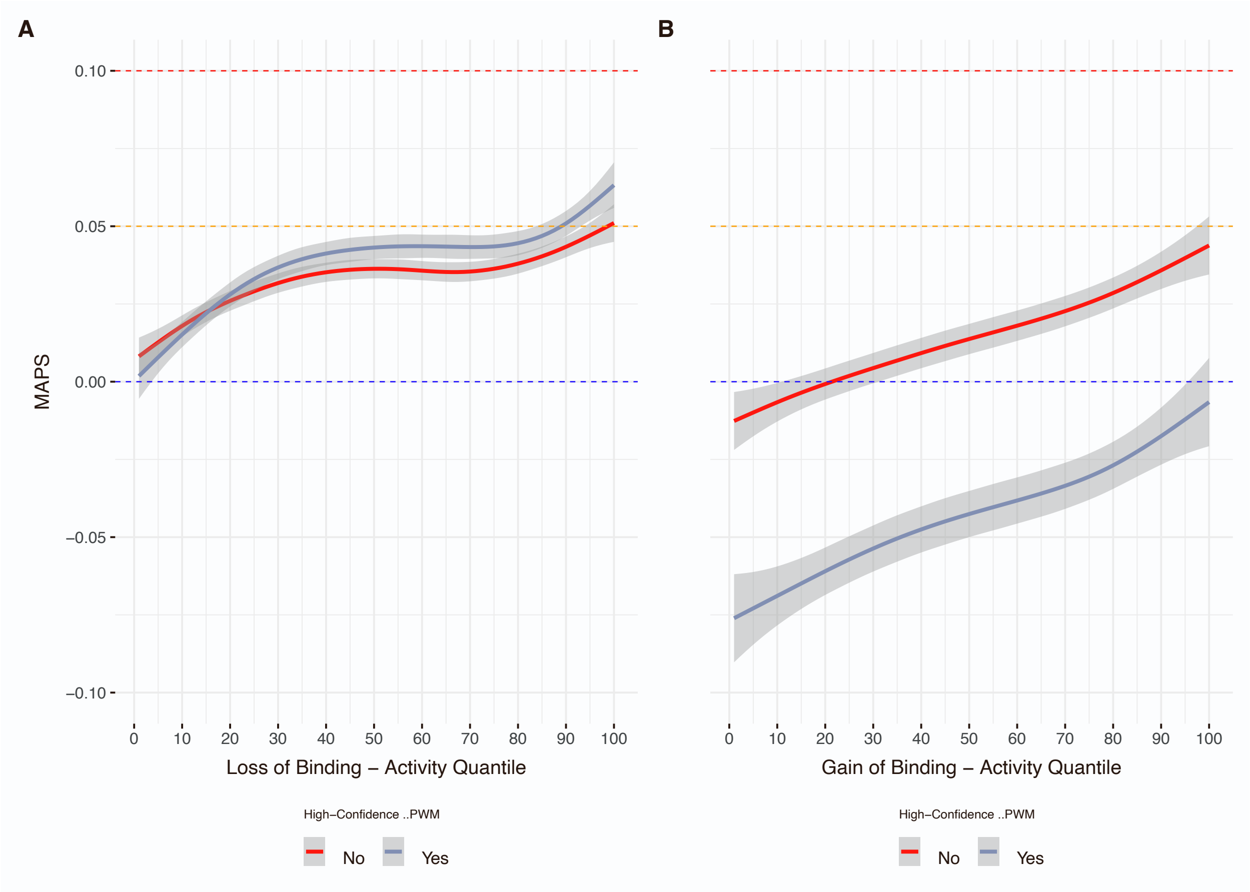

Figure S6. A) Relationship between allele frequency as measured by MAPS and the loss of CTCF binding activity stratified by confidence in the ΔPWM calls. For context, we display MAPS scores for synonymous (blue line), missense (orange) and splicing (red) variants. **B)** Relationship between allele frequency as measured by MAPS and the gain of CTCF binding activity stratified by confidence in the ΔPWM calls. A-B) Plotted are smoothed conditional means using the LOESS method.

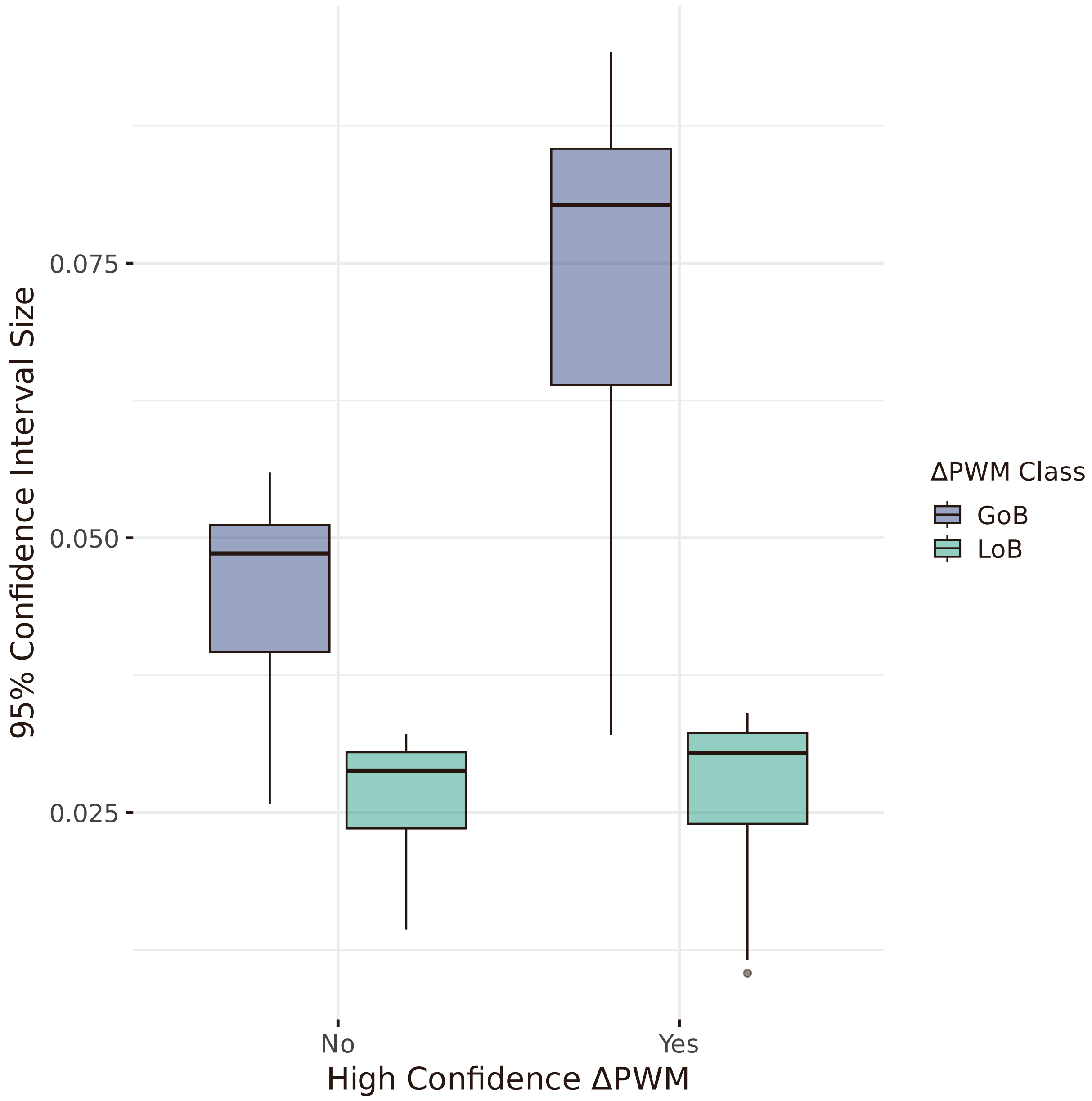

Figure S7. Size of 95% confidence intervals on all singleton proportions used to calculate MAPS scores. Confidence was measured by applying a binomial test on the proportion of singletons variants in each binding activity quantile, stratified by the confidence in each variant's ΔPWM score. Gain of binding and loss of binding are abbreviated as GoB and LoB, respectively.

Annotation	N	Length (bp)	Footprint (Mb)	Coverage Conservation	Coverage CADD	Number SNVs
rDHS	1063878	273	290	--	--	--
rDHS + PWM	355418	15	5	100	0.24	1253229

Table S1. Summary statistics of the study dataset. Statistics describing the noncoding sequence footprint of putative CBSs identified through our approach, stratified by whether the CBS has both rDHS and PWM support. The fields are as follows; N is the number of sequence elements in each group, Length is the mean length of all sequences for a given group in basepairs, footprint is the cumulative amount of genomic sequence covered through each annotation approach, coverage conservation describes the proportion of sequence annotated with evolutionary sequence conservation (GERP++, LINSIGHT, PhyloP100 and Phastcons100), Coverage CADD is the proportion of sequence annotated with CADD scores and Number of SNVs refers to the total number of overlapping SNVs in gnomAD. Dashed lines (--) signal data we did not collect as a part of this study.

Variant Class	N	MAPS	SEM
Synonymous	2926343	0.0000	0.0003
3’ UTR	10000000	0.0275	0.0002
Intergenic	19999998	0.0286	0.0001
Missense	6075537	0.0435	0.0002
5’ UTR	9442976	0.0558	0.0002
Splice Donor	112806	0.0965	0.0015
Start Lost	26198	0.0985	0.0030
Stop Lost	12784	0.1122	0.0043
Stop Gained	201570	0.1196	0.0011
Splice Acceptor	82838	0.1256	0.0017

Table S2. MAPS scores for different functional classes of genic variation in gnomAD. Error was calculated as the standard error of the mean for each proportion. Variant class indicates the Variant Effect Predictor (VEP) worst consequence. The count of 3’UTR and Intergenic variants were limited to a random sample of the displayed size to facilitate their processing.
