# Supplementary material for: Identifying deleterious noncoding variation through gain and loss of CTCF binding activity
Source: Am J Hum Genet. 2025 Mar 5;112(4):892–902. doi: 10.1016/j.ajhg.2025.02.009 (PMC12081274; doi:10.1016/j.ajhg.2025.02.009)
Supplement: Document S2. Article plus supplemental information [file mmc2.pdf]

# Identifying deleterious noncoding variation through gain and loss of CTCF binding activity


## Authors

Colby Tubbs, Mary Lauren Benton, Evonne McArthur, John A. Capra, Douglas M. Ruderfer

## Correspondence

colby.a.tubbs@vanderbilt.edu (C.T.), douglas.ruderfer@vumc.org (D.M.R.)



**This study develops a functional annotation scheme for CTCF binding site (CBS) variants and applies it to 76,156 WGS samples in gnomAD. In these data, we find evidence for purifying selection acting against the loss of CTCF binding, supporting a functional role for CBS variation in human traits and disease.**



Tubbs et al., 2025, The American Journal of Human Genetics *112*, 892–902
April 3, 2025

© 2025 The Author(s). Published by Elsevier Inc. on behalf of American Society of Human Genetics.

https://doi.org/10.1016/j.ajhg.2025.02.009

ARTICLE

# Identifying deleterious noncoding variation through gain and loss of CTCF binding activity

Colby Tubbs,[1,*] Mary Lauren Benton,[2] Evonne McArthur,[3] John A. Capra,[4] and Douglas M. Ruderfer[1,5,*]


## Summary

CCCTC binding factor (CTCF) regulates gene expression through DNA binding at thousands of genomic loci. Genetic variation in these CTCF binding sites (CBSs) is an important driver of phenotypic variation, yet extracting those that are likely to have functional consequences in whole-genome sequencing remains challenging. To address this, we develop a hypothesis-driven framework to identify and prioritize CBS variants in gnomAD. We synthesize CTCF's binding patterns at 1,063,878 genomic loci across 214 biological contexts into a summary of binding activity. We find that high binding activity significantly correlates with both conserved nucleotides (Pearson $R = 0.35$, $p < 2.2 \times 10^{-16}$) and sequences that contain high-quality CTCF binding motifs (Pearson $R = 0.63$, $p = 2.9 \times 10^{-12}$). We then use binding activity to evaluate high-confidence allelic binding predictions for 1,253,329 single-nucleotide variations (SNVs) in gnomAD that disrupt a CBS. We find a strong, positive relationship between the mutability-adjusted proportion of singletons (MAPS) metric and the loss of CTCF binding at loci with high *in vitro* activity (Pearson $R = 0.74$, $p < 2.2 \times 10^{-16}$). To contextualize these findings, we apply MAPS to other functional classes of variation and find that a subset of 339,380 loss of CTCF binding variants is observed as infrequently as missense variants are. This work nominates these thousands of rare, noncoding variants that disrupt CTCF binding for further functional studies while providing a blueprint for prioritizing variation in other transcription factor binding sequences.


## Introduction

CCCTC binding factor (CTCF) functions by binding its DNA sequence motif at thousands of genomic loci.[1,2] These CTCF binding sites (CBSs) can facilitate or repress gene expression by modulating the genome's three-dimensional (3D) organization.[3–6] Genetic variation that disrupts CBSs is an important contributor to phenotypic variation; CBS variants occur frequently in cancers[7,8] and, in rare instances, factor in pathogenic gene expression underlying Mendelian disorders.[9,10] Despite their clear importance, only a small number of CBS variants have been well characterized, and there is currently little consensus on the functional impact of CBS disruption in human populations.[11–18] The systematic prioritization of CBS variation remains challenging given the complexity around identifying active CTCF binding sequences and accurately predicting which genetic variants will disrupt their function. Nonetheless, a comprehensive and large-scale characterization of CBS variation would provide a means to directly evaluate the extent to which CBS variants mediate risk for disease and thus could prove an invaluable resource for the community.

Previous functional studies have profiled CBS variation in both monogenic and complex diseases. Focused investigations into genetic variation at the *EPHA4*, 17q2, and *ITIH3* loci have demonstrated the disruption of CTCF binding in risk for a spectrum of rare limb malformities, asthma, and schizophrenia, respectively.[9,19,20] At the *EPHA4* and 17q2 loci, the alteration of one or more CBSs drives the disorganization of topologically associated domains (TADs), which, in turn, induces aberrant patterns of gene expression underlying the disease phenotype. Alternatively, small-scale sequencing efforts have broadly targeted CBSs by profiling single-nucleotide variation (SNVs) that disrupt either the CTCF motif or binding peaks from chromatin immunoprecipitation sequencing (ChIP-seq). These studies have found a burden of CBS variation in complex traits, including numerous cancer subtypes and Alzheimer disease.[8,21] While these studies are constrained to specific phenotypes and small sample sizes, together they suggest an important role for CBS variation in disease architecture.

Several larger-scale surveys that include CBS variation have found mixed evidence for their functional roles in disease pathology. When partitioning complex trait heritability by functional annotation, Finucane et al.[17] found no significant enrichment of variation in CTCF ChIP footprints. More recently, Reshef et al.[18] extended this methodology to discover significant relationships between genome-wide association study (GWAS) SNPs disruptive of CBSs and several complex traits, including eczema and lupus. Furthermore, Han et al.[16] observed low allele frequencies (AFs) associated with the loss of CTCF binding through copy-number variation in several large-scale whole-genome sequencing (WGS) datasets. Collectively, these data paint an intriguing but complicated picture of the functional impact of CBS variants in human populations.

[1]Division of Genetic Medicine, Department of Medicine, Vanderbilt Genetics Institute, Vanderbilt University Medical Center, Nashville, TN, USA; [2]Department of Computer Science, Baylor University, Waco, TX, USA; [3]Department of Medicine, University of Washington, Seattle, WA, USA; [4]Department of Epidemiology and Biostatistics, Bakar Computational Health Sciences Institute, University of California, San Francisco, San Francisco, CA, USA; [5]Center for Digital Genomic Medicine, Vanderbilt University Medical Center, Nashville, TN, USA
*Correspondence: colby.a.tubbs@vanderbilt.edu (C.T.), douglas.ruderfer@vumc.org (D.M.R.)
https://doi.org/10.1016/j.ajhg.2025.02.009.
© 2025 The Author(s). Published by Elsevier Inc. on behalf of American Society of Human Genetics.
This is an open access article under the CC BY license (http://creativecommons.org/licenses/by/4.0/).

Prioritizing CBS variants at scale is not straightforward, given the difficulty in detecting active CBSs at base-pair resolution and annotating variants without the guidance of a genetic code.[22,23] ChIP-seq directly assays CTCF binding but reports peaks that span hundreds of base pairs[24] and requires potentially thousands of biosamples to fully map CTCF's cell-type-specific binding preferences.[4] Alternatively, CBSs can be defined as motifs and detected through precision weight matrices[25] (PWMs). PWMs, however, detect binding based on the contribution of DNA sequences only and, therefore, suffer from an enormous false positive rate (i.e., the motif futility theorem[26]). Although these modalities can be integrated,[27] there is no defined framework to convert the binding patterns they detect across cell types into a useful annotation for variants. Finally, extracting which variants are likely to have functional effects on transcription factor (TF) binding *in vivo* remains challenging. While numerous allelic binding predictors exist,[28–31] they are primarily benchmarked on predicting chromatin features or affinity changes *in vitro* and have not been evaluated on their ability to prioritize deleterious variations.

To address these challenges, we propose an analytical framework for the large-scale identification and prioritization of CBS variation in WGS. In our approach, we first define CBSs as motifs that fall within regions profiled for DNase and CTCF activity across hundreds of biological contexts by leveraging recently available data from ENCODE's registry of candidate *cis*-regulatory elements (cCREs).[32] To convert CTCF's cross-cell-type binding patterns at these loci into a functional annotation, we quantify them into a summary metric, which we call binding activity. Next, we use binding activity to provide important context to measurements of allelic binding for over 1 million SNVs in gnomAD v.3, which consists of 76,156 publicly available WGS samples.[33] We then evaluate these annotations by comparing them to metrics of conservation, deleteriousness, and evidence for negative selection.

In applying our framework, we identify CBS variations at scale and provide a comprehensive evaluation of constraint on those that are predicted to meaningfully alter CBS activity. Using orthogonal data, we validate the utility of binding activity in detecting CBSs enriched for likely functional consequences. We then detect strong signatures of negative selection acting on thousands of variants that are rare and predicted to induce the loss of CTCF binding at loci with robust binding activity. In summary, we provide a catalog of prioritized CBS variants in human populations that, ultimately, will enable the improved interpretation of genetic contributions to human disorders.

## Material and methods

### Annotating rDHSs with CTCF binding activity

We downloaded the CTCF $Z$ score signal matrix for all representative DNase hypersensitivity sites (rDHSs) from the ENCODE-SCREEN web portal.[32,34] We subset this matrix to rDHSs with a significant DNase signal in at least one biosample, which corresponds to the complete set of human cCREs ($n = 1,063,878$). We masked all rDHS-biosample combinations with a raw CTCF binding signal of zero ($Z$ score equal to $-10$). To generate the binding activity annotation, we summarized each rDHS's CTCF signal distribution using Stouffer's meta-analysis of $Z$ scores.[35] To do this, we implemented the equation $\Sigma zi/N$, where $zi$ represents a given biosample's $Z$ score and $N$ is the total number of non-masked biosamples for that rDHS.

### Annotating CTCF motifs with binding activity and definition of activity quantiles

We downloaded all reference (REF) sequence matches to CTCF's canonical motif (MA0139.2) in build hg38 from the JASPAR database.[36] We then excluded sequence matches from non-autosomal and sex contigs, reported assembly gaps, ENCODE blacklisted regions,[37] and protein-coding exons (Gencode v.44[38]) using Bedtools *intersect*.[39] Our final track consisted of 1,870,772 sequence matches. To integrate these data with rDHS regions from ENCODE (above), we intersected the subset of rDHSs that are cCREs with our final set of motifs using Bedtools *intersect*. We assigned each motif an activity score by reporting the activity of its intersecting cCRE. In the case of multiple overlaps (i.e., bookended cCREs), we report the cCRE with the higher activity score. Motifs that did not intersect a cCRE were not assigned activity scores. In certain analyses, we refer to binding activity in terms of quantiles. To do this, we assigned the distribution of activity scores for all annotated rDHSs into 100 equal-sized bins.

### Comparing predicted CTCF binding affinity with activity

We reported each CTCF motif's predicted binding affinity as its PWM score, relative to the minimum and maximum scores obtainable as in Gheorghe et al.[27] To characterize the relationship between the binding activity and these relative PWM scores, we binned each motif by their activity quantile (above) and assessed the mean relative PWM score in each bin. Their statistical evaluation was computed using a Pearson correlation.

### Comparing evolutionary sequence conservation with CTCF binding activity

We assessed nucleotide conservation using conservation metrics.[40–42] PhyloP100, PhastCons100, and GERP++ scores were downloaded in Bigwig format from the UCSC Genome Browser.[43] LINSIGHT scores were downloaded from Huang et al.[44] We assigned all positions corresponding to motifs with rDHS support conservation scores using the package pyBigWig.[45] In the case of conservation scores generated in build hg19 (GERP++ and LINSIGHT), we first lifted back each position's coordinate using the UCSC LiftOver tool.[46] To assess the relative enrichment of conserved nucleotides against binding activity, we calculated the proportion of conserved nucleotides in each activity decile. We used thresholds of 2, 0.8, 0.8, and 1 to define conserved positions for GERP++, LINSIGHT, PhastCons100, and Phylop100, respectively. To assess the confidence of each calculation, we randomly sampled with replacement nucleotides from each activity quantile 10,000 times over 10 iterations. We plotted the mean of these computations with their corresponding 95% confidence interval. We assessed the relationship and statistical significance using Spearman correlation.

### Processing genetic data from gnomAD

We downloaded the gnomAD v.3.1.2 database and reduced it to SNVs, removing entries with an allele count (AC) and AF reported as zero. We further required all SNVs to be of high quality (quality control [QC] = PASS) and covered in at least half of the samples in gnomAD v.3 (allele number [AN] > 76,000). In total, we identified 569,860,911 SNVs after QC. For each variant, we report its functional class as its most severe consequence term as assessed by the Variant Effect Predictor[47] (VEP).

### Annotating gain and loss of binding for all candidate SNVs in gnomAD

First, we identified all QC'd SNVs that overlap the subset of CTCF binding motifs with cCRE support using Bedtools *intersect*. To calculate ΔPWM scores and accompanying $p$ values, we used the R package atSNP.[48,49] To do this, we extracted 14 bp of the flanking REF sequences upstream and downstream of each variant's genomic coordinate. We then report the highest log-odds score for the subsequence overlapping the variant's position for both the REF and alternate (ALT) alleles assessed by atSNP. Raw ΔPWM scores were quantified as the difference between the REF and ALT PWM scores. We classify variants as putative gain of binding if their raw ΔPWM was $\leq 0$ and putative loss of binding if their raw ΔPWM was $> 0$. To assess the significance of each observed ΔPWM score, we reported its –log10 $p$ value rank score, which is derived from atSNP's importance sampling algorithm. To address truncation errors related to low $p$ values ($p = \sim 0$), we encoded dummy values of ($10^{-7}$).

### Assessing constraint using scaled CADD scores

We extracted PHRED-scaled combined annotation-dependent depletion (CADD)[50] scores for each variant from the gnomAD v.3.1.2 Hail table. To assess the relationship between these scores, binding activity, and ΔPWM, we first stratified all variants by their class (gain of binding or loss of binding) and then by their significance ($p < 0.05$). For each binding activity decile, we randomly sampled variants with replacement 10,000 times over 10 iterations. For each iteration, we calculated the proportion of variants that are putatively pathogenic using a scaled CADD cutoff of 10. We plot the mean of these calculations and 95% confidence intervals.

### Implementing MAPS scores

We implemented mutability-adjusted proportion of singletons (MAPS) scores as described in Karzewski et al.[51] using custom scripts as follows. To annotate all SNVs in gnomAD with their trinucleotide mutation rate, we first identified their trinucleotide context by reporting each nucleotide upstream and downstream of the variant's position using the package pySAM.[52] Next, we downloaded trinucleotide mutation rate data from the gnomAD v.2 flagship paper[51] and merged it with each variant's trinucleotide context. To calibrate the MAPS model, we used all QC'd SNVs with their most severe VEP annotation as "synonymous_variant" ($n = 2{,}161{,}831$). We then referred to this trained model to predict MAPS for all other functional variant classes. For a given class, we calculated a raw singleton proportion as the number of singletons (AC = 1) divided by the number of variants. We then applied the calibrated model to regress out the expected number of singletons within that bin given its average mutation rate. To assess confidence, we computed the standard error of the mean (SEM) for each bin.

## Results

### Framework for the functional annotation of CBS variants

We present a framework to catalog all likely functional CBS variants in gnomAD (Figure 1). To achieve this, we sought to discover all possible CBSs in the genome using CTCF's PWM and experimental binding data. We first detected all REF genome sequence matches to CTCF's PWM ($N = 1{,}870{,}772$; Figure S1A; material and methods). To extract motif hits likely to function as CBSs *in vivo*, we overlapped them with 1,063,878 of ENCODE's representative DNase hypersensitivity regions (rDHSs) (material and methods). Nineteen percent ($n = 355{,}418$) of CTCF's motifs fall within an annotated rDHS (Figure S1B), regardless of the cCRE classification. Conversely, 290,793 (27%) of rDHSs contained at least 1 CTCF motif, while most contained fewer than 4 (Figure S1C). We henceforth refer to the intersection of these data (i.e., CTCF motifs with rDHS overlap, $n = 355{,}418$) simply as CBSs (Table S1).

At each rDHS, ENCODE has estimated the strength of CTCF binding as a $Z$ score relative to all loci tested in each cell line or tissue sample (i.e., biosample) (Figure 1A). These data are used to call CTCF binding in the cCRE framework by thresholding on a $Z$ score of >1.64. We sought to evaluate the sensitivity of CBS calling at the rDHS containing a CTCF motif ($n = 290{,}793$) to this thresholding. We observed that modest changes to the detection threshold resulted in a substantial difference in the number of rDHSs called as "CTCF bound" (Figure S1D). We further observed that, at the minimal threshold used to define CTCF binding in the ENCODE framework, a substantial proportion of binding events were observed in only a single biosample (Figure S1D).

### Developing a summary annotation of CTCF's binding activity across biological contexts

We observed considerable variability in CBS calling when thresholding on CTCF's ChIP-seq $Z$ score and that, when assessing all profiled biosamples, many CBSs fail to replicate. We thus sought to quantify the strength and replication of CTCF's activity at each CBS into a single metric (similar to an intolerance metric for a gene[53–55]). CTCF's binding activity was summarized at each rDHS by meta-analyzing its distribution of binding activity $Z$ scores across all biosamples (mean = 2.58, SD = 10.14; Figure 2A; material and methods). For simplicity, we refer to this annotation as binding activity.

To validate the use of binding activity in differentiating CBSs with robust activity, we quantified its relationship with characteristics that are likely to signal authentic binding sequences. We note that we evaluate binding activity by summarizing characteristics across its distribution in the form of quantiles (material and methods; Figure S2). Each quantile held an average of 3,554 CBSs and 53,312 nucleotides. First, we observed an enrichment

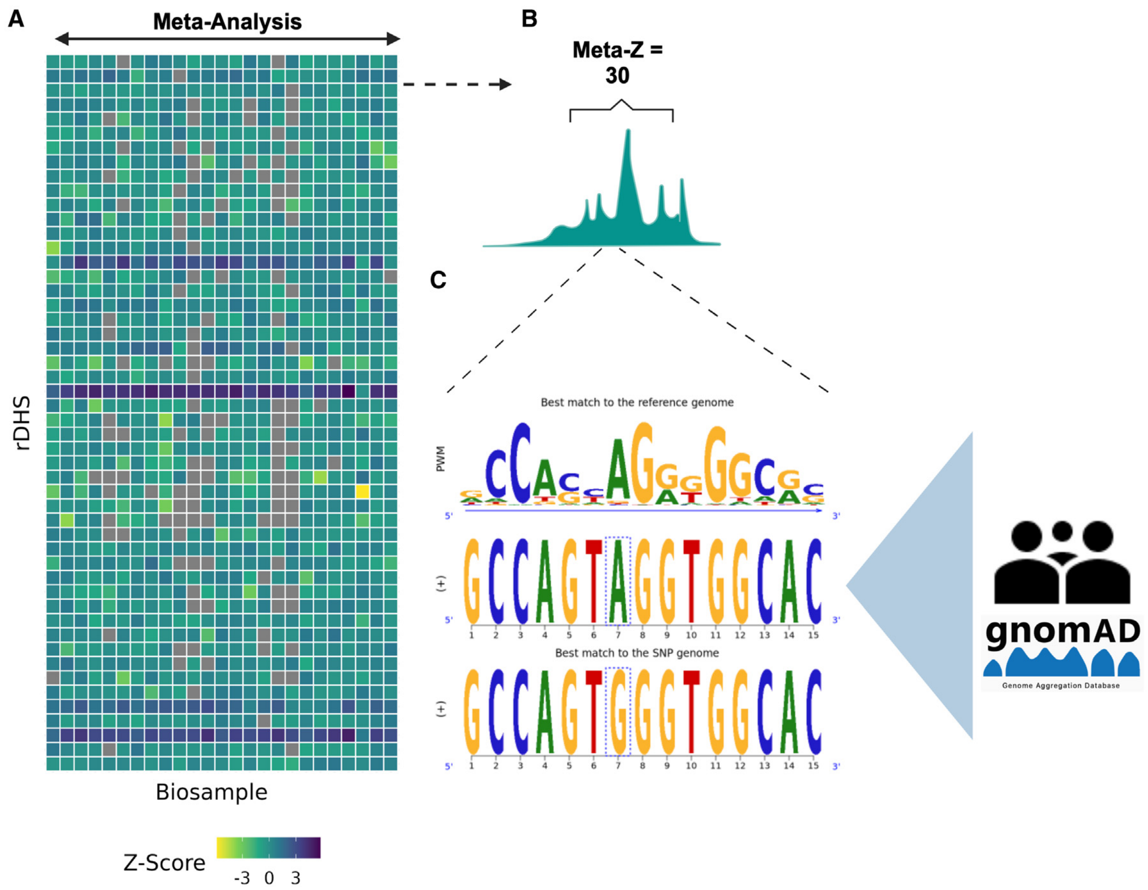


**Figure 1. Schematic overview of the study approach**
(A) Heatmap of CTCF's ChIP-seq binding activity *Z* score at selected rDHSs and biosamples. The x axis represents 25 of 214 randomly sampled biosamples, and the y axis represents 100 of 1,063,879 randomly sampled rDHSs. Coloring corresponds to the strength of binding (*Z* score) for a given biosample and rDHS.
(B) Schematic illustration of a theoretical distribution of binding activity *Z* scores for a single rDHS across 25 biosamples. We utilize these data to develop an annotation for CTCF's observed binding patterns across these biosamples, which we refer to as binding activity. Here, binding activity would be quantified as a single summary score by meta-analyzing the depicted distribution of *Z* scores using Stouffer's method.[35]
(C) Schematic illustrating the integration of binding activity with the functional annotation of SNVs through ΔPWM scores. Pictured as an example is a putative loss-of-binding SNV that has reduced binding energy in the alternate allele. We calculate high-confidence ΔPWM scores using atSNP's importance sampling technique for all candidate SNVs in 76,156 WGS samples from gnomAD.

of CBSs occurring in high quantiles of binding activity (Pearson $R = 0.70$, $p < 2.8 \times 10^{-16}$; Figure 2B). We then quantified each CBS's predicted binding affinity for CTCF through its PWM (material and methods) and observed a strong correlation between binding activity quantiles and predicted binding energy (Pearson $R = 0.63$, $p = 2.9 \times 10^{-12}$; Figure 2C). Second, we evaluated the relationship between binding activity quantiles and functional constraint by annotating greater than 99% of the 5.3 Mb of CTCF binding sequence with evolutionary conservation metrics. PhlyoP scores quantify conservation at single nucleotides, making them well suited for assessing constraint at high resolution. We calculated the proportion of conserved PhlyoP scores for each binding activity quantile and observed significantly higher proportions of conserved positions belonging to motifs in higher binding activity quantiles (Pearson $R = 0.35$, $p < 2.2 \times 10^{-16}$; Figure 2D). Although conservation scores vary in their methodology, this correlation was consistent across multiple metrics (Figure S3).

## Integrating binding activity with variant-level allelic binding predictions in gnomAD

Next, we sought to combine our binding activity annotation with variant-level measurements of allelic binding. First, we identified all SNVs in gnomAD that disrupt a CBS ($N = 1,253,229$; material and methods). For each SNV in our dataset, we calculated its change from REF PWM (ΔPWM) and classified it as putative gain or loss of binding (material and methods). We observed fewer gain-of-binding SNVs ($n = 238,196$) compared to loss-of-binding SNVs ($n = 1,015,133$) (Figure 3A). To assess the confidence of each observed ΔPWM score, we calculated each score's test statistic by implementing the importance sampling procedure developed in the atSNP package[48,49] (material and methods). We identified high-confidence

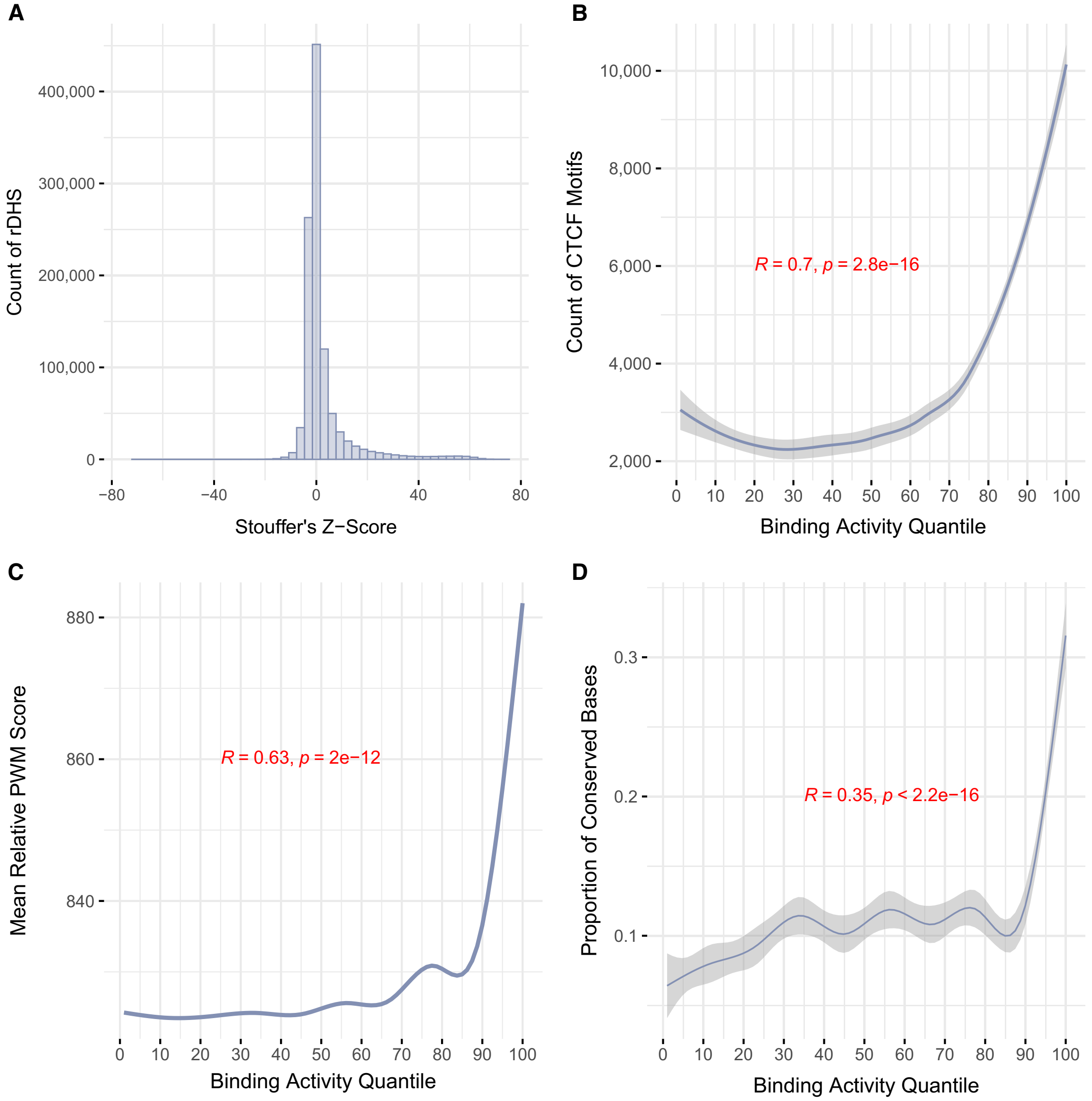


**Figure 2. CTCF binding activity identifies sequence enriched for functional signatures**
(A) Distribution of meta-analyzed $Z$ scores for all 1,063,878 rDHSs used in this study. Meta-analysis was conducted using Stouffer's method.
(B) The frequency of overlap between high-quality CTCF motifs and all rDHSs belonging to a given quantile of binding activity. Quantiles were created by placing meta-analyzed $Z$ scores for all rDHSs from (A) into 100 equal-sized bins.
(C) The mean PWM score for all CTCF motifs that overlap an rDHS in each quantile of binding activity.
(D) Relationship between evolutionary sequence conservation of CTCF motif sequence and binding activity quantiles. Conservation was measured as the proportion of conserved CTCF motif positions within each quantile using PhlyoP100 scores. A score threshold of 1 was used to define conserved positions.
(B–D) Pearson correlation and the accompanying $p$ value was used for statistical evaluation. Plotted are smoothed conditional means using the LOESS method.[56,57]

gain-of-binding ($n$ = 63,931) and loss-of-binding ($n$ = 527,785) SNVs using a significance threshold of $p < 0.05$ (Figure 3B). We then integrated these predictions with binding activity by assigning each variant its motif's activity score (Figure 3C; material and methods). We observed significantly more high-confidence loss- and gain-of-binding SNVs in high quantiles of binding activity (loss-of-binding Pearson $R = 0.65$, $p = 2.7 \times 10^{-13}$; gain-of-binding Pearson $R = 0.67$, $p = 2.1 \times 10^{-14}$).

## Characterizing signatures of selection on the loss of CTCF binding activity

Deleterious genetic variants are purged from populations by natural selection, which enables the use of AF as an informative metric for the utility of an annotation scheme in capturing functional variants.[58,59] For example, SNVs that disrupt genes are observed more rarely than those in intergenic space, while splicing SNVs are observed more rarely than those that fall in introns.[51] We evaluated

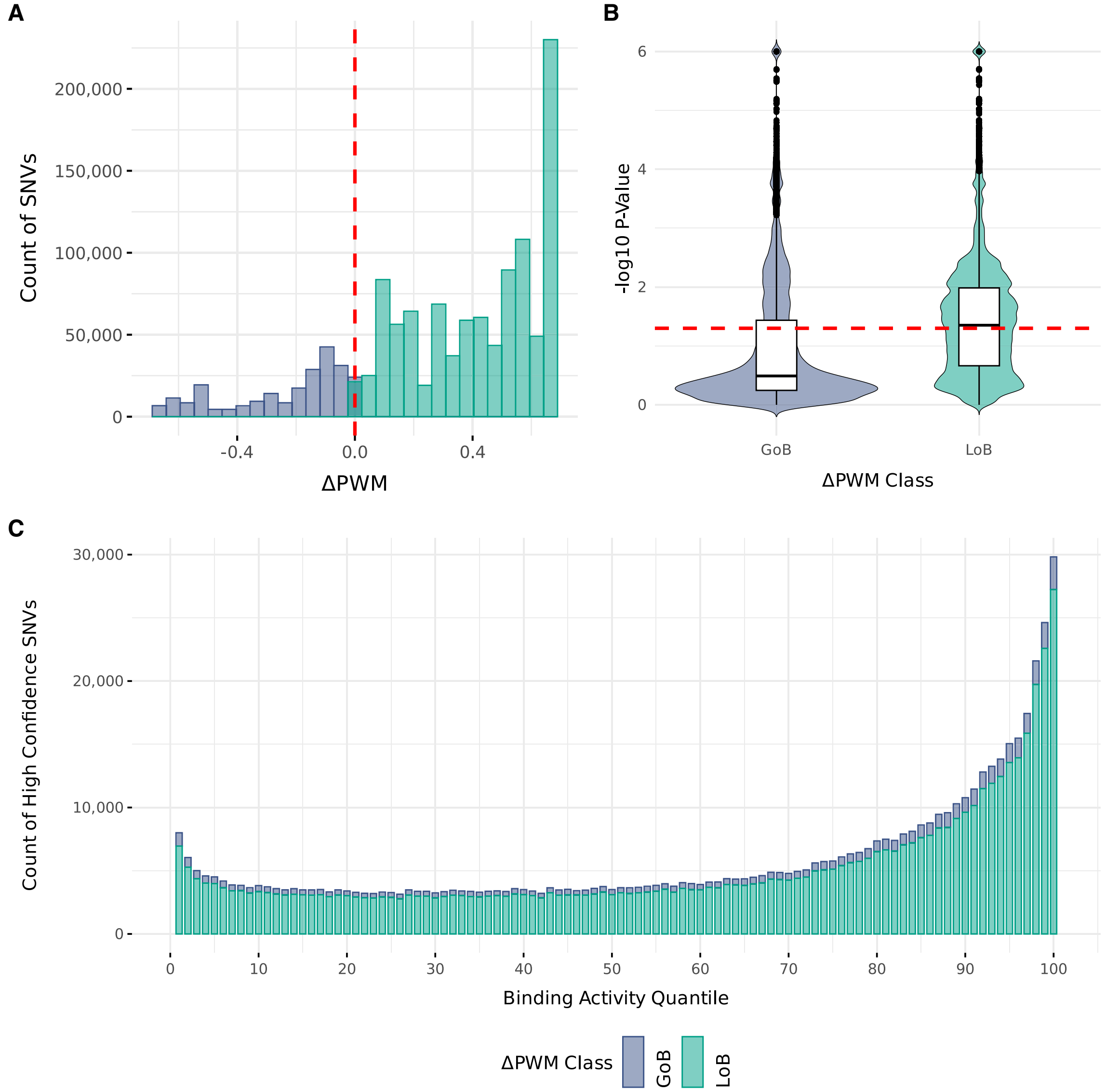


**Figure 3. Integrating binding activity with ΔPWM to functionally annotate CBS variants in gnomAD**
(A) Distribution of ΔPWM scores for all SNVs in gnomAD that disrupt a CTCF motif with rDHS support. Gain-of-binding (GoB) variants were considered those that have a ΔPWM of $\leq 0$. Loss-of-binding (LoB) variants are defined as those having a ΔPWM of $>0$.
(B) Distributions of ΔPWM test statistics for all SNVs in (A) assessed through atSNP's importance sampling procedure. Truncated $p$ values (approximately 0) were manually encoded as $10^{-5}$. Red line indicates the threshold used ($p < 0.05$) for distinguishing low- and high-confidence ΔPWM scores.
(C) Counts of high-confidence ΔPWM scores in gnomAD for SNVs that disrupt a CTCF motif with rDHS support, binned by their binding activity quantile. Counts are stratified by their ΔPWM class (GoB and LoB).

our annotation approach in this context by quantifying the relationship between variants identified in our framework with different measures of selective constraint. To do this, we applied two measures: PHRED-scaled CADD[50] scores and the MAPS metric. First, we observed significantly higher proportions of predicted pathogenic variants (scaled CADD $\geq 10$, Pearson $R = 0.53$, $p < 2.2 \times 10^{-16}$) for SNVs annotated as high-confidence loss of binding activity (Figure 4A). To assess the contribution of statistical testing on ΔPWM scores to this result, we calculated the proportion of predicted pathogenic variants for each binding activity quantile after stratifying variants by confidence in their ΔPWM score. At the 95$^{th}$ quantile of activity or higher, we observed nominally higher frequencies of predicted pathogenic variants for high-confidence ΔPWM scores compared to low-confidence scores (high-confidence ΔPWM mean proportion pathogenic $= 0.33$, low-confidence ΔPWM mean $= 0.26$, Student's t test, $p = 1.83 \times 10^{-2}$; Figure S4A). We observed no significant difference between low- and high-confidence ΔPWM scores for gain-of-binding SNVs in this analysis (Figure S4B).

Second, we assessed the raw AFs for all CBS variants and observed that they are exceedingly rare in gnomAD (Figure S5). We then quantified this by calculating MAPS scores for all SNVs in each activity quantile, stratified by

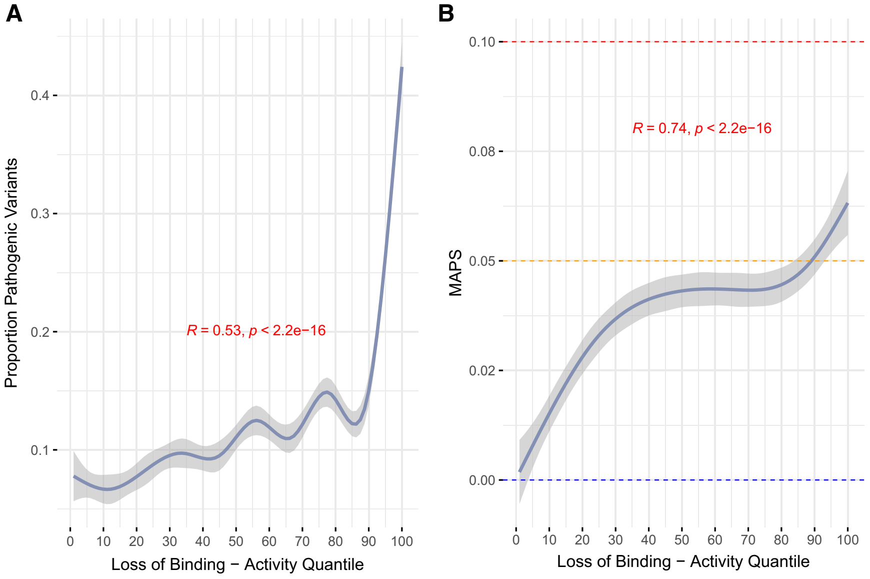


**Figure 4. Loss of CTCF binding activity is under high levels of constraint in gnomAD**
(A) Relationship between proportion of putative pathogenic SNVs based on PHRED-scaled CADD scores and the loss of CTCF binding activity. A scaled CADD score of ≥10 was used as a threshold of pathogenic or not. The y axis displays the proportion of pathogenic SNVs within each activity quantile. Error is shown as a 95% bootstrapped confidence interval.
(B) Relationship between allele frequency as measured by MAPS and the loss of CTCF binding activity. For context, we display MAPS scores for synonymous (blue line), missense (orange), and splicing (red) variants in gnomAD.
(A and B) Pearson correlation and the accompanying $p$ value was used for statistical evaluation. Plotted are smoothed conditional means using the LOESS method.

their predicted effect on binding affinity and the level of confidence in their ΔPWM scores. The loss of CTCF binding activity correlated significantly with MAPS, with a stronger overall effect for high-confidence SNVs (high-confidence Pearson $R = 0.74$, $p = 2.2 \times 10^{-16}$, Figures 4B and S6A). We note that while gain of binding at both high and low ΔPWM confidence displayed higher MAPS scores at higher levels of activity (Figure S6B), there are substantially fewer SNVs in each bin. Consequently, we observe higher uncertainty on individual MAPS scores for both high- and low-confidence gain-of-binding SNVs (Figure S7). To contextualize our findings, we calculated MAPS for different functional classes of intergenic and genic SNVs in gnomAD (Table S2). Loss-of-binding SNVs in CBS with low activity trended toward MAPS scores of likely neutral classes of variation (i.e., synonymous and intergenic), regardless of the confidence in the ΔPWM call. High-impact loss-of-binding variants (activity decile ≥ 90) had higher MAPS scores than missense variants (MAPS loss of binding of activity decile 90 = 0.05, MAPS missense = 0.04).

## Discussion

Expanded use of genome sequencing combined with large-scale assays of regulatory function are providing opportunities for the systematic characterization of noncoding SNVs. Here, we leverage these data to quantify the functional impact of CBS variation in a population of over 76,156 WGS samples. We show that the loss of CTCF binding activity is associated with high levels of constraint and occurs infrequently in human populations. In applying our approach, we generated a catalog of functionally prioritized CBS variants while providing a scalable blueprint for the interpretable annotation of TF binding site variants.

ENCODE's cCRE dataset and the widespread availability of PWMs have enabled the extensive mapping of CTCF binding sequence across cellular contexts. The remaining challenge has been leveraging these multidimensional data to effectively annotate variants. Our approach assigns CTCF motifs with an aggregate measure of binding activity across 214 biological contexts. We show the utility of binding activity in several ways. First, we found that high binding activity correlates with functional signatures, including evolutionary conservation. This result was robust across multiple conservation metrics and suggests that binding activity effectively captures sequences that are likely to have functional consequences. Second, we show that the context provided by binding activity is necessary for the effective interpretation of allelic binding predictions; we observed that high-confidence ΔPWM scores in CBS with low binding activity resemble likely neutral classes of variation (e.g., synonymous) in terms of their frequency in the population. Several databases have collectively assembled billions of ΔPWM scores by measuring SNVs against PWMs in an all-against-all framework.[28,48] Our results here suggest that a substantial proportion of these are likely false positives and that further annotation efforts should, when possible, contextualize their measurements with functional assays of binding activity.

We observed strong signals of negative selection on SNVs that induce the loss of CTCF binding activity. Intriguingly, variants with the highest impact on binding activity had higher MAPS scores than missense variants. While the role of protein alterations in disease etiologies is well established, we are only beginning to understand the functional consequences of CBS disruption. CBSs have a critical function in 3D genome organization. For example, accumulating evidence suggests that properly bound and oriented CBSs are crucial for the formation of

TADs through loop extrusion.[60,61] In other contexts, densely bound neighborhoods of CBSs in a similar binding orientation may produce an insulatory effect by separating TADs.[62] Variants may disrupt binding at these chromatin loop anchors or TAD boundaries, thereby driving gene misexpression through 3D genome disorganization.[63–67] Further functional characterization of the CBSs implicated in our study will be an invaluable approach to extend the mechanistic interpretation of high-impact variants.

We note several important limitations in our findings. Our annotation approach requires the uniform processing and integration of a TF's binding patterns through ChIP-seq across potentially thousands of biosamples. Currently, this depth of data exists only for a few TFs, like CTCF.[68,69] We anticipate the ability to annotate more TFs and therefore more SNVs as these data expand. Furthermore, our summary of these binding patterns into a metric of activity is an effective means to distinguish likely functional binding sequences, which is critical for interpreting genetic variants. However, it does not incorporate tissue-specific activity and may down-weight CBSs that are highly active in poorly profiled tissues. Additionally, we note the bias inherent in thresholding for high-quality motifs when integrating CTCF's PWM with binding activity. It remains unclear as to whether this bias contributed to the identification of substantially more loss-of-binding SNVs in gnomAD or if there are important differences in the functional properties of gain-of-binding SNVs. Finally, our approach does not annotate variants through a single score but rather assesses their impact through the combination of two annotations: binding activity and ΔPWM class. Determining the best way to weight these metrics in a single summary score is unclear but remains a promising future direction.

We apply our approach specifically to CBS variation but expect it has potential for future expansion to other TFs. Our framework consists of 3 components: large-scale genetic data (gnomAD), experimental binding annotations, and PWMs. While CTCF is the only TF with direct integration in the cCRE dataset, binding data for many other proteins exist and could be curated in a similar fashion. Furthermore, PWMs are widely available for hundreds of TFs[36] and, as we demonstrate, offer a straightforward integration with ChIP peaks. Despite the availability of more complex models, PWMs remain ideal for large-scale variant annotation given their simplicity and low-computational costs. We therefore expect broad utility for our framework in the future annotation of noncoding variants.

We note that many noncoding variant annotators exist, including those both agnostic and specific to TF binding variation.[28–31,68,70–73] For example, CADD produces pathogenicity scores based on a variant's relationship to many genomic annotations, while others, like DeepSea and CATO, explicitly predict allelic binding. Utilizing these methods to catalog likely functional variation in CBS, as we do here, was unclear. CADD is precomputed for the entire genome but provides little biological insight into the variants measured, while allelic binding predictors, like DeepSea, require a set of input variants *a priori*. Defining which CBSs and variants to target is unclear without the additional information we provide in our approach (i.e., binding activity). Finally, we expect that binding activity may benefit from integration with machine learning outputs, but it remains challenging to evaluate given the lack of gold-standard functional data on CBS variants.

In summary, we present a framework to prioritize CBS variants that capitalizes on both the large population genome-sequencing datasets and extensive gene regulatory annotations that now exist. We demonstrate a conceptually and computationally tractable strategy to synthesize these data and, in doing so, find evidence for purifying selection acting against the loss of CTCF binding in human populations. We anticipate that this catalog will aid in the identification of functional CBS variants while providing a critical foundation for the future annotation of noncoding variation.

## Data and code availability

All code used for constructing the study dataset, conducting analysis, and generating the figures is available as a Snakemake workflow in the Ruderfer Lab CBS_FunctionalAnnotation GitHub repository: https://github.com/RuderferLab/CBS_FunctionalAnnotation. The repository also contains two summary files detailing activity scores and ΔPWM scores for all CBS and variants evaluated in the study.


## Acknowledgments

This work was funded by the National Institute of Mental Health (NIMH) and grant ID: R01MH123155.


## Declaration of interests

The authors declare no competing interests.

## Supplemental information

Supplemental information can be found online at https://doi.org/10.1016/j.ajhg.2025.02.009.

## Web resources

CompGen, http://compgen.cshl.edu/LINSIGHT/LINSIGHT.bw

ENCODE-SCREEN, https://downloads.wenglab.org/cCREs/matrices/all/GRCh38.CTCF-zscore.rDHS-V3.txt.gz

gnomAD, https://gnomad.broadinstitute.org/data

JASPAR, https://jaspar.elixir.no/api/v1/matrix/MA0139.2.jaspar

UCSC Genome Browser, GERP++ scores, https://hgdownload.soe.ucsc.edu/gbdb/hg19/bbi/All_hg19_RS.bw

UCSC Genome Browser, Phastcons100, http://hgdownload.cse.ucsc.edu/goldenpath/hg38/phastCons100way

UCSC Genome Browser, PhyloP100, http://hgdownload.cse.ucsc.edu/goldenpath/hg38/phylop100way

Received: September 4, 2024
Accepted: February 10, 2025
Published: March 5, 2025

## References

1. Essien, K., Vigneau, S., Apreleva, S., Singh, L.N., Bartolomei, M.S., and Hannenhalli, S. (2009). CTCF binding site classes exhibit distinct evolutionary, genomic, epigenomic and transcriptomic features. Genome Biol. *10*, R131.
2. Kim, T.H., Abdullaev, Z.K., Smith, A.D., Ching, K.A., Loukinov, D.I., Green, R.D., Zhang, M.Q., Lobanenkov, V.V., and Ren, B. (2007). Analysis of the Vertebrate Insulator Protein CTCF-Binding Sites in the Human Genome. Cell *128*, 1231–1245.
3. Huang, H., Zhu, Q., Jussila, A., Han, Y., Bintu, B., Kern, C., Conte, M., Zhang, Y., Bianco, S., Chiariello, A.M., et al. (2021). CTCF mediates dosage- and sequence-context-dependent transcriptional insulation by forming local chromatin domains. Nat. Genet. *53*, 1064–1074.
4. Ong, C.T., and Corces, V.G. (2014). CTCF: An architectural protein bridging genome topology and function. Nat. Rev. Genet. *15*, 234–246.
5. Phillips, J.E., and Corces, V.G. (2009). CTCF: Master Weaver of the Genome. Cell *137*, 1194–1211.
6. Rao, S.S.P., Huntley, M.H., Durand, N.C., Stamenova, E.K., Bochkov, I.D., Robinson, J.T., Sanborn, A.L., Machol, I., Omer, A.D., Lander, E.S., and Aiden, E.L. (2014). A 3D map of the human genome at kilobase resolution reveals principles of chromatin looping. Cell *159*, 1665–1680.
7. Fang, C., Wang, Z., Han, C., Safgren, S.L., Helmin, K.A., Adelman, E.R., Serafin, V., Basso, G., Eagen, K.P., Gaspar-Maia, A., et al. (2020). Cancer-specific CTCF binding facilitates oncogenic transcriptional dysregulation. Genome Biol. *21*, 247.
8. Katainen, R., Dave, K., Pitkänen, E., Palin, K., Kivioja, T., Välimäki, N., Gylfe, A.E., Ristolainen, H., Hänninen, U.A., Cajuso, T., et al. (2015). CTCF/cohesin-binding sites are frequently mutated in cancer. Nat. Genet. *47*, 818–821.
9. Lupiáñez, D.G., Kraft, K., Heinrich, V., Krawitz, P., Brancati, F., Klopocki, E., Horn, D., Kayserili, H., Opitz, J.M., Laxova, R., et al. (2015). Disruptions of topological chromatin domains cause pathogenic rewiring of gene-enhancer interactions. Cell *161*, 1012–1025.
10. Spielmann, M., Lupiáñez, D.G., and Mundlos, S. (2018). Structural variation in the 3D genome. Nat. Rev. Genet. *19*, 453–467.
11. Azazi, D., Mudge, J.M., Odom, D.T., and Flicek, P. (2020). Functional signatures of evolutionarily young CTCF binding sites. BMC Biol. *18*, 132.
12. Xu, B., Wang, H., Wright, S., Hyle, J., Zhang, Y., Shao, Y., Niu, M., Fan, Y., Rosikiewicz, W., Djekidel, M.N., et al. (2021). Acute depletion of CTCF rewires genome-wide chromatin accessibility. Genome Biol. *22*, 244.
13. Hyle, J., Zhang, Y., Wright, S., Xu, B., Shao, Y., Easton, J., Tian, L., Feng, R., Xu, P., and Li, C. (2019). Acute depletion of CTCF directly affects MYC regulation through loss of enhancer–promoter looping. Nucleic Acids Res. *47*, 6699–6713.
14. Nora, E.P., Goloborodko, A., Valton, A.-L., Gibcus, J.H., Uebersohn, A., Abdennur, N., Dekker, J., Mirny, L.A., and Bruneau, B.G. (2017). Targeted Degradation of CTCF Decouples Local Insulation of Chromosome Domains from Genomic Compartmentalization. Cell *169*, 930–944.e22.
15. Hsieh, T.-H.S., Cattoglio, C., Slobodyanyuk, E., Hansen, A.S., Darzacq, X., and Tjian, R. (2022). Enhancer–promoter interactions and transcription are largely maintained upon acute loss of CTCF, cohesin, WAPL or YY1. Nat. Genet. *54*, 1919–1932.
16. Han, L., Zhao, X., Benton, M.L., Perumal, T., Collins, R.L., Hoffman, G.E., Johnson, J.S., Sloofman, L., Wang, H.Z., Stone, M.R., et al. (2020). Functional annotation of rare structural variation in the human brain. Nat. Commun. *11*, 2990.
17. Finucane, H.K., Bulik-Sullivan, B., Gusev, A., Trynka, G., Reshef, Y., Loh, P.R., Anttila, V., Xu, H., Zang, C., Farh, K., et al. (2015). Partitioning heritability by functional annotation using genome-wide association summary statistics. Nat. Genet. *47*, 1228–1235.
18. Reshef, Y.A., Finucane, H.K., Kelley, D.R., Gusev, A., Kotliar, D., Ulirsch, J.C., Hormozdiari, F., Nasser, J., O'Connor, L., Van De Geijn, B., et al. (2018). Detecting genome-wide directional effects of transcription factor binding on polygenic disease risk. Nat. Genet. *50*, 1483–1493.
19. Li, Y., Ma, C., Li, S., Wang, J., Li, W., Yang, Y., Li, X., Liu, J., Yang, J., Liu, Y., et al. (2022). Regulatory Variant rs2535629 in *ITIH3* Intron Confers Schizophrenia Risk By Regulating CTCF Binding and *SFMBT1* Expression. Adv. Sci. *9*, 2104786.
20. Schmiedel, B.J., Seumois, G., Samaniego-Castruita, D., Cayford, J., Schulten, V., Chavez, L., Ay, F., Sette, A., Peters, B., and Vijayanand, P. (2016). 17q21 asthma-risk variants switch CTCF binding and regulate IL-2 production by T cells. Nat. Commun. *7*, 13426.
21. Patel, P.J., Ren, Y., and Yan, Z. (2023). Epigenomic analysis of Alzheimer's disease brains reveals diminished CTCF binding on genes involved in synaptic organization. Neurobiol. Dis. *184*, 106192.
22. Singh, V., Crick, F.H.C., Leslie Barnett, F.R.S., Brenner, S., and Watts-Tobin, R.J. (2019). General Nature of the Genetic Code for Proteins. Reson. *24*, 1171–1178.
23. French, J.D., and Edwards, S.L. (2020). The Role of Noncoding Variants in Heritable Disease. Trends Genet. *36*, 880–891.
24. Furey, T.S. (2012). ChIP-seq and beyond: New and improved methodologies to detect and characterize protein-DNA interactions. Nat. Rev. Genet. *13*, 840–852.
25. Stormo, G.D. (2013). Modeling the specificity of protein-DNA interactions. Quant. Biol. *1*, 115–130.
26. Wasserman, W.W., and Sandelin, A. (2004). Applied bioinformatics for the identification of regulatory elements. Nat. Rev. Genet. *5*, 276–287.
27. Gheorghe, M., Sandve, G.K., Khan, A., Chèneby, J., Ballester, B., and Mathelier, A. (2019). A map of direct TF–DNA interactions in the human genome. Nucleic Acids Res. *47*, 7715.
28. Steinhaus, R., Robinson, P.N., and Seelow, D. (2022). FABIAN-variant: predicting the effects of DNA variants on transcription factor binding. Nucleic Acids Res. *50*, W322–W329.
29. Kelley, D.R., Snoek, J., and Rinn, J.L. (2016). Basset: learning the regulatory code of the accessible genome with deep convolutional neural networks. Genome Res. *26*, 990–999.
30. Maurano, M.T., Haugen, E., Sandstrom, R., Vierstra, J., Shafer, A., Kaul, R., and Stamatoyannopoulos, J.A. (2015). Large-scale identification of sequence variants influencing human transcription factor occupancy in vivo. Nat. Genet. *47*, 1393–1401.
31. Zhou, J., and Troyanskaya, O.G. (2015). Predicting effects of noncoding variants with deep learning–based sequence model. Nat. Methods *12*, 931–934.
32. Abascal, F., Acosta, R., Addleman, N.J., Adrian, J., Afzal, V., Aken, B., Akiyama, J.A., Jammal, O.A., Amrhein, H.,

Anderson, S.M., et al. (2020). Expanded encyclopaedias of DNA elements in the human and mouse genomes. Nature *583*, 699–710.
33. Chen, S., Francioli, L.C., Goodrich, J.K., Collins, R.L., Kanai, M., Wang, Q., Alföldi, J., Watts, N.A., Vittal, C., Gauthier, L.D., et al. (2024). A genomic mutational constraint map using variation in 76,156 human genomes. Nature *625*, 92–100.
34. Luo, Y., Hitz, B.C., Gabdank, I., Hilton, J.A., Kagda, M.S., Lam, B., Myers, Z., Sud, P., Jou, J., Lin, K., et al. (2020). New developments on the Encyclopedia of DNA Elements (ENCODE) data portal. Nucleic Acids Res. *48*, D882–D889.
35. Chang, L.-C., Lin, H.-M., Sibille, E., and Tseng, G.C. (2013). Meta-analysis methods for combining multiple expression profiles: comparisons, statistical characterization and an application guideline. BMC Bioinf. *14*, 368.
36. Rauluseviciute, I., Riudavets-Puig, R., Blanc-Mathieu, R., Castro-Mondragon, J.A., Ferenc, K., Kumar, V., Lemma, R.B., Lucas, J., Chèneby, J., Baranasic, D., et al. (2024). JASPAR 2024: 20th anniversary of the open-access database of transcription factor binding profiles. Nucleic Acids Res. *52*, D174–D182.
37. Amemiya, H.M., Kundaje, A., and Boyle, A.P. (2019). The ENCODE Blacklist: Identification of Problematic Regions of the Genome. Sci. Rep. *9*, 9354.
38. Frankish, A., Carbonell-Sala, S., Diekhans, M., Jungreis, I., Loveland, J.E., Mudge, J.M., Sisu, C., Wright, J.C., Arnan, C., Barnes, I., et al. (2023). GENCODE: reference annotation for the human and mouse genomes in 2023. Nucleic Acids Res. *51*, D942–D949.
39. Quinlan, A.R., and Hall, I.M. (2010). BEDTools: A flexible suite of utilities for comparing genomic features. Bioinformatics *26*, 841–842.
40. Felsenstein, J., and Churchill, G.A. (1996). A Hidden Markov Model approach to variation among sites in rate of evolution. Mol. Biol. Evol. *13*, 93–104.
41. Siepel, A., Bejerano, G., Pedersen, J.S., Hinrichs, A.S., Hou, M., Rosenbloom, K., Clawson, H., Spieth, J., Hillier, L.W., Richards, S., et al. (2005). Evolutionarily conserved elements in vertebrate, insect, worm, and yeast genomes. Genome Res. *15*, 1034–1050.
42. Pollard, K.S., Hubisz, M.J., Rosenbloom, K.R., and Siepel, A. (2010). Detection of nonneutral substitution rates on mammalian phylogenies. Genome Res. *20*, 110–121.
43. Nassar, L.R., Barber, G.P., Benet-Pagès, A., Casper, J., Clawson, H., Diekhans, M., Fischer, C., Gonzalez, J.N., Hinrichs, A.S., Lee, B.T., et al. (2023). The UCSC Genome Browser database: 2023 update. Nucleic Acids Res. *51*, D1188–D1195.
44. Huang, Y.-F., Gulko, B., and Siepel, A. (2017). Fast, scalable prediction of deleterious noncoding variants from functional and population genomic data. Nat. Genet. *49*, 618–624.
45. Ramírez, F., Dündar, F., Diehl, S., Grüning, B.A., and Manke, T. (2014). deepTools: a flexible platform for exploring deep-sequencing data. Nucleic Acids Res. *42*, W187–W191.
46. Hinrichs, A.S., Karolchik, D., Baertsch, R., Barber, G.P., Bejerano, G., Clawson, H., Diekhans, M., Furey, T.S., Harte, R.A., Hsu, F., et al. (2006). The UCSC Genome Browser Database: update 2006. Nucleic Acids Res. *34*, D590–D598.
47. McLaren, W., Gil, L., Hunt, S.E., Riat, H.S., Ritchie, G.R.S., Thormann, A., Flicek, P., and Cunningham, F. (2016). The Ensembl Variant Effect Predictor. Genome Biol. *17*, 122.
48. Shin, S., Hudson, R., Harrison, C., Craven, M., and Keleş, S. (2019). atSNP Search: a web resource for statistically evaluating influence of human genetic variation on transcription factor binding. Bioinformatics *35*, 2657–2659.
49. Zuo, C., Shin, S., and Keleş, S. (2015). atSNP: transcription factor binding affinity testing for regulatory SNP detection. Bioinformatics *31*, 3353–3355.
50. Rentzsch, P., Witten, D., Cooper, G.M., Shendure, J., and Kircher, M. (2019). CADD: predicting the deleteriousness of variants throughout the human genome. Nucleic Acids Res. *47*, D886–D894.
51. Karczewski, K.J., Francioli, L.C., Tiao, G., Cummings, B.B., Alföldi, J., Wang, Q., Collins, R.L., Laricchia, K.M., Ganna, A., Birnbaum, D.P., et al. (2020). The mutational constraint spectrum quantified from variation in 141,456 humans. Nature *581*, 434–443.
52. Danecek, P., Bonfield, J.K., Liddle, J., Marshall, J., Ohan, V., Pollard, M.O., Whitwham, A., Keane, T., McCarthy, S.A., Davies, R.M., and Li, H. (2021). Twelve years of SAMtools and BCFtools. GigaScience *10*, giab008.
53. Lek, M., Karczewski, K.J., Minikel, E.V., Samocha, K.E., Banks, E., Fennell, T., O'Donnell-Luria, A.H., Ware, J.S., Hill, A.J., Cummings, B.B., et al. (2016). Analysis of protein-coding genetic variation in 60,706 humans. Nature *536*, 285–291.
54. Havrilla, J.M., Pedersen, B.S., Layer, R.M., and Quinlan, A.R. (2019). A map of constrained coding regions in the human genome. Nat. Genet. *51*, 88–95.
55. Petrovski, S., Wang, Q., Heinzen, E.L., Allen, A.S., and Goldstein, D.B. (2013). Genic Intolerance to Functional Variation and the Interpretation of Personal Genomes. PLoS Genet. *9*, e1003709.
56. Cleveland, W.S., and Devlin, S.J. (1988). Locally Weighted Regression: An Approach to Regression Analysis by Local Fitting. J. Am. Stat. Assoc. *83*, 596–610.
57. Cleveland, W.S. (1979). Robust Locally Weighted Regression and Smoothing Scatterplots. J. Am. Stat. Assoc. *74*, 829–836.
58. Short, P.J., McRae, J.F., Gallone, G., Sifrim, A., Won, H., Geschwind, D.H., Wright, C.F., Firth, H.V., Fitzpatrick, D.R., Barrett, J.C., and Hurles, M.E. (2018). De novo mutations in regulatory elements in neurodevelopmental disorders. Nature *555*, 611–616.
59. Lord, J., Gallone, G., Short, P.J., McRae, J.F., Ironfield, H., Wynn, E.H., Gerety, S.S., He, L., Kerr, B., Johnson, D.S., et al. (2019). Pathogenicity and selective constraint on variation near splice sites. Genome Res. *29*, 159–170.
60. Li, Y., Haarhuis, J.H.I., Sedeño Cacciatore, Á., Oldenkamp, R., Van Ruiten, M.S., Willems, L., Teunissen, H., Muir, K.W., De Wit, E., Rowland, B.D., and Panne, D. (2020). The structural basis for cohesin–CTCF-anchored loops. Nature *578*, 472–476.
61. Fudenberg, G., Imakaev, M., Lu, C., Goloborodko, A., Abdennur, N., and Mirny, L.A. (2016). Formation of Chromosomal Domains by Loop Extrusion. Cell Rep. *15*, 2038–2049.
62. Nanni, L., Ceri, S., and Logie, C. (2020). Spatial patterns of CTCF sites define the anatomy of TADs and their boundaries. Genome Biol. *21*, 197.
63. Northcott, P.A., Lee, C., Zichner, T., Stütz, A.M., Erkek, S., Kawauchi, D., Shih, D.J.H., Hovestadt, V., Zapatka, M., Sturm, D., et al. (2014). Enhancer hijacking activates GFI1 family oncogenes in medulloblastoma. Nature *511*, 428–434.
64. Gröschel, S., Sanders, M.A., Hoogenboezem, R., de Wit, E., Bouwman, B.A.M., Erpelinck, C., van der Velden, V.H.J., Havermans, M., Avellino, R., van Lom, K., et al. (2014). A Single Oncogenic Enhancer Rearrangement Causes Concomitant EVI1 and GATA2 Deregulation in Leukemia. Cell *157*, 369–381.

65. Greenwald, W.W., Li, H., Benaglio, P., Jakubosky, D., Matsui, H., Schmitt, A., Selvaraj, S., D'Antonio, M., D'Antonio-Chronowska, A., Smith, E.N., and Frazer, K.A. (2019). Subtle changes in chromatin loop contact propensity are associated with differential gene regulation and expression. Nat. Commun. *10*, 1054.
66. McArthur, E., and Capra, J.A. (2021). Topologically associating domain boundaries that are stable across diverse cell types are evolutionarily constrained and enriched for heritability. Am. J. Hum. Genet. *108*, 269–283.
67. Despang, A., Schöpflin, R., Franke, M., Ali, S., Jerković, I., Paliou, C., Chan, W.L., Timmermann, B., Wittler, L., Vingron, M., et al. (2019). Functional dissection of the Sox9–Kcnj2 locus identifies nonessential and instructive roles of TAD architecture. Nat. Genet. *51*, 1263–1271.
68. Dunham, I., Kundaje, A., Aldred, S.F., Collins, P.J., Davis, C.A., Doyle, F., Epstein, C.B., Frietze, S., Harrow, J., Kaul, R., et al. (2012). An integrated encyclopedia of DNA elements in the human genome. Nature *489*, 57–74.
69. Ziebarth, J.D., Bhattacharya, A., and Cui, Y. (2013). CTCFBSDB 2.0: a database for CTCF-binding sites and genome organization. Nucleic Acids Res. *41*, D188–D194.
70. Shihab, H.A., Rogers, M.F., Gough, J., Mort, M., Cooper, D.N., Day, I.N.M., Gaunt, T.R., and Campbell, C. (2015). An integrative approach to predicting the functional effects of non-coding and coding sequence variation. Bioinformatics *31*, 1536–1543.
71. Ritchie, G.R.S., Dunham, I., Zeggini, E., and Flicek, P. (2014). Functional annotation of noncoding sequence variants. Nat. Methods *11*, 294–296.
72. Quang, D., Chen, Y., and Xie, X. (2015). DANN: A deep learning approach for annotating the pathogenicity of genetic variants. Bioinformatics *31*, 761–763.
73. Kircher, M., Witten, D.M., Jain, P., O'roak, B.J., Cooper, G.M., and Shendure, J. (2014). A general framework for estimating the relative pathogenicity of human genetic variants. Nat. Genet. *46*, 310–315.

The American Journal of Human Genetics, Volume *112*

## Supplemental information

# Identifying deleterious noncoding variation

# through gain and loss of CTCF binding activity

**Colby Tubbs, Mary Lauren Benton, Evonne McArthur, John A. Capra, and Douglas M. Ruderfer**

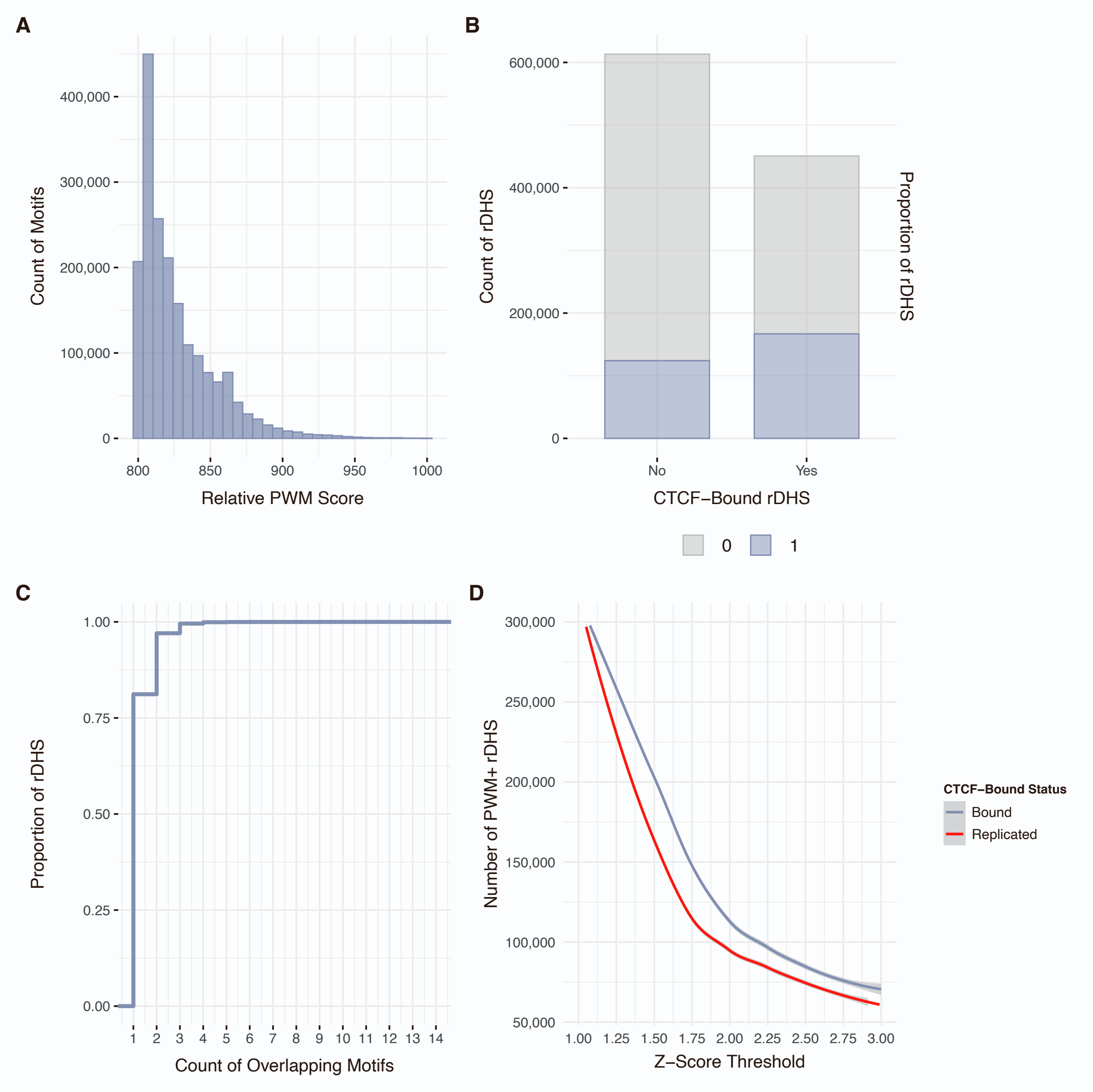


**Figure S1. PWMs and ChIP-Seq alone are insufficient for the robust detection of active CTCF binding sequence. A)** Distribution of binding energies for all high-quality sequence matches (N = 1,870,772) to the canonical CTCF PWM (JASPAR MA0139.2) in the hg38 reference genome. The threshold for detection was a PWM score of 80% or higher relative to the max possible score for the CTCF PWM. **B)** Counts of rDHS stratified by whether the rDHS is classified as "CTCF Bound" using criteria from ENCODE (Z-score > 1.64 in at least biosample). Each group is colored to reflect the proportion of rDHS that have >=1 overlapping CTCF binding motif. **C)** Cumulative proportion of rDHS by the number of overlapping CTCF motifs after reducing to rDHS with >=1 overlapping motif. **D)** Count of rDHS that would be called as CTCF-bound given the provided Z-score threshold. The "Bound" status requires binding in any of the 214 profiled biosamples. "Replicated" refers to CTCF binding replicated in > 1 biosample. rDHS were subset to those containing at least 1 CTCF PWM match. Plotted are smoothed conditional means using the LOESS method.

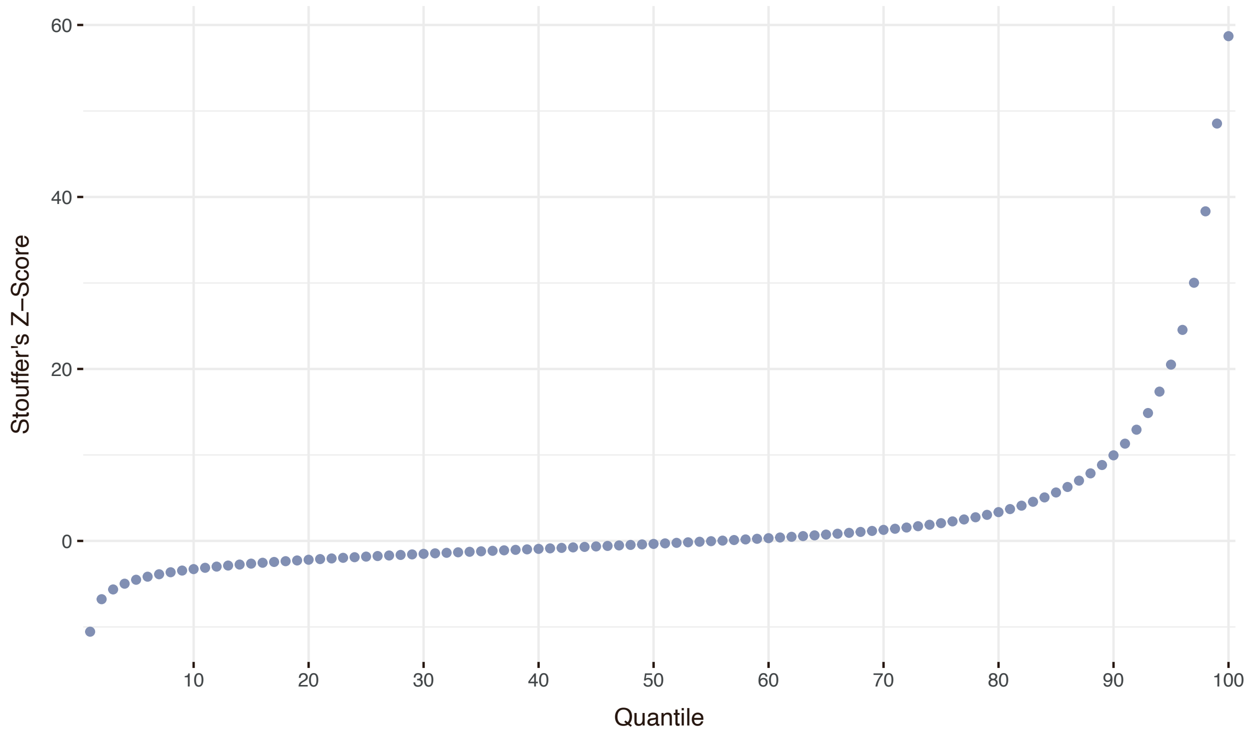


**Figure S2. Overview of meta-analyzed Z-scores for each quantile of binding activity.** Distributions of meta-analyzed binding activity scores after binning all rDHS into 100 equal sized bins. Each quantile contains approximately 10,638 unique rDHS.

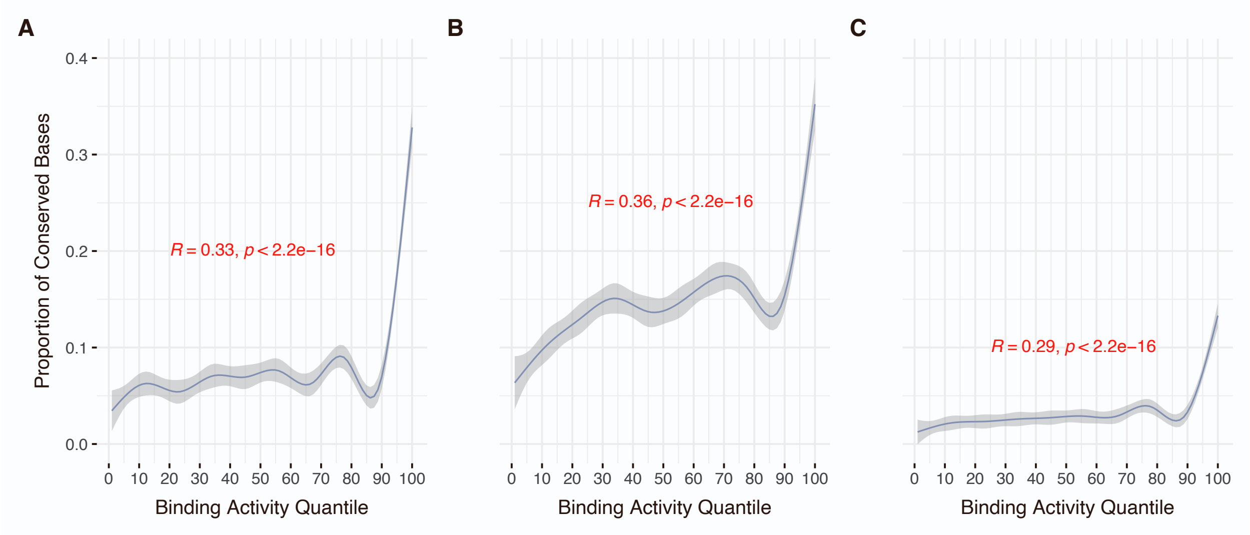


**Figure S3. The enrichment of evolutionary sequence conservation at high binding activity quantiles is robust across different conservation metrics.** Relationship between evolutionary sequence conservation of CTCF motifs and binding activity quantiles. Conservation was measured as a proportion of conserved CTCF motif positions within each quantile using GERP++, LINSIGHT and Phastcons100 scores. Conservation was measured as a proportion of conserved bases within each bin using a threshold of 2, 0.8 and 0.8 for GERP++, LINSIGHT and Phastcons100 respectively. A-C) Plotted are smoothed conditional means using the LOESS method.

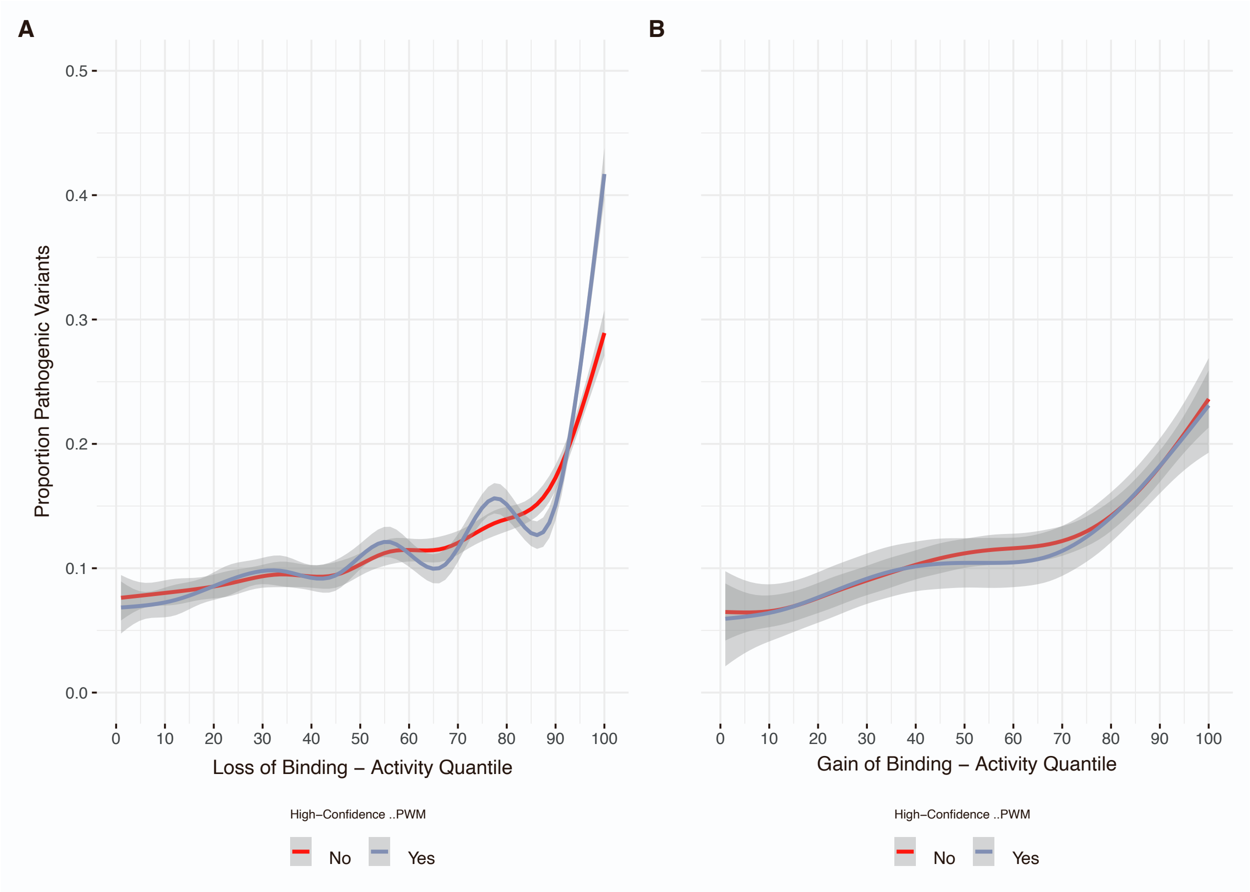


**Figure S4. A)** Relationship between proportion of putative pathogenic SNVs based on scaled CADD scores and the loss of CTCF binding activity. A scaled CADD score of >=10 was used as a threshold of pathogenic or not. Y-axis displays the proportion of pathogenic SNVs within each activity quantile. Error is plotted as a 95% bootstrapped confidence interval. Colors indicate stratification on the confidence of the ΔPWM call using a threshold of 0.05. **B)** Relationship between the proportion of putative pathogenic SNVs based on scaled CADD scores and the gain of CTCF binding activity assessed using the same approach as in A. A-B) Plotted are smoothed conditional means using the LOESS method.

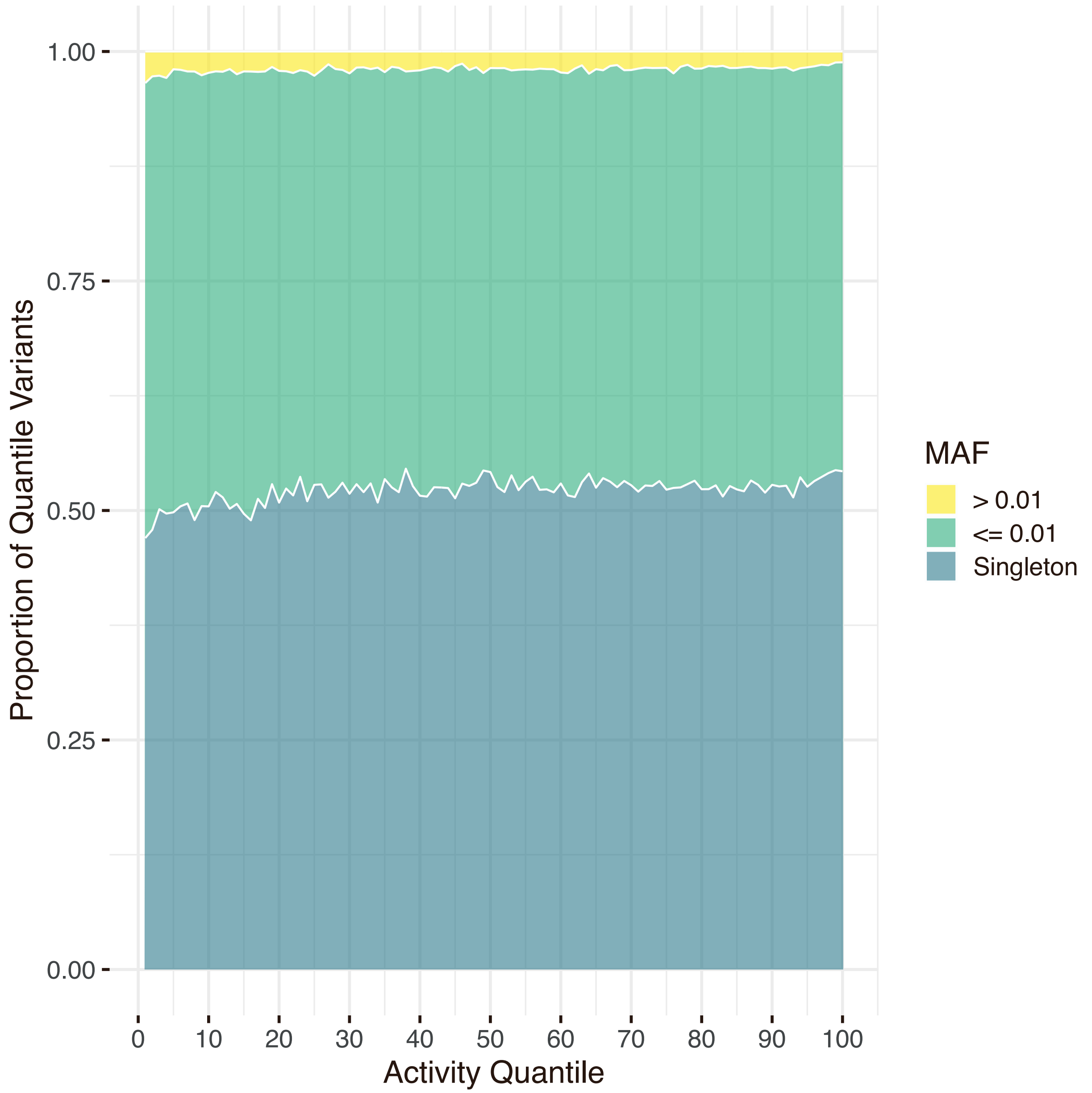


**Figure S5.** Allele frequency overview of all (n=527,785) significant loss of binding SNVs in gnomAD that disrupt CBSs. These variants represent a subset of all variants identified in the study. Variants were classified as singletons (allele count of 1), MAF <= 0.01, or MAF > 0.01. The X-axis depicts these variants binned by their binding activity quantile. The Y-axis represents the proportion of variants in each quantile belonging to a given allele frequency class.

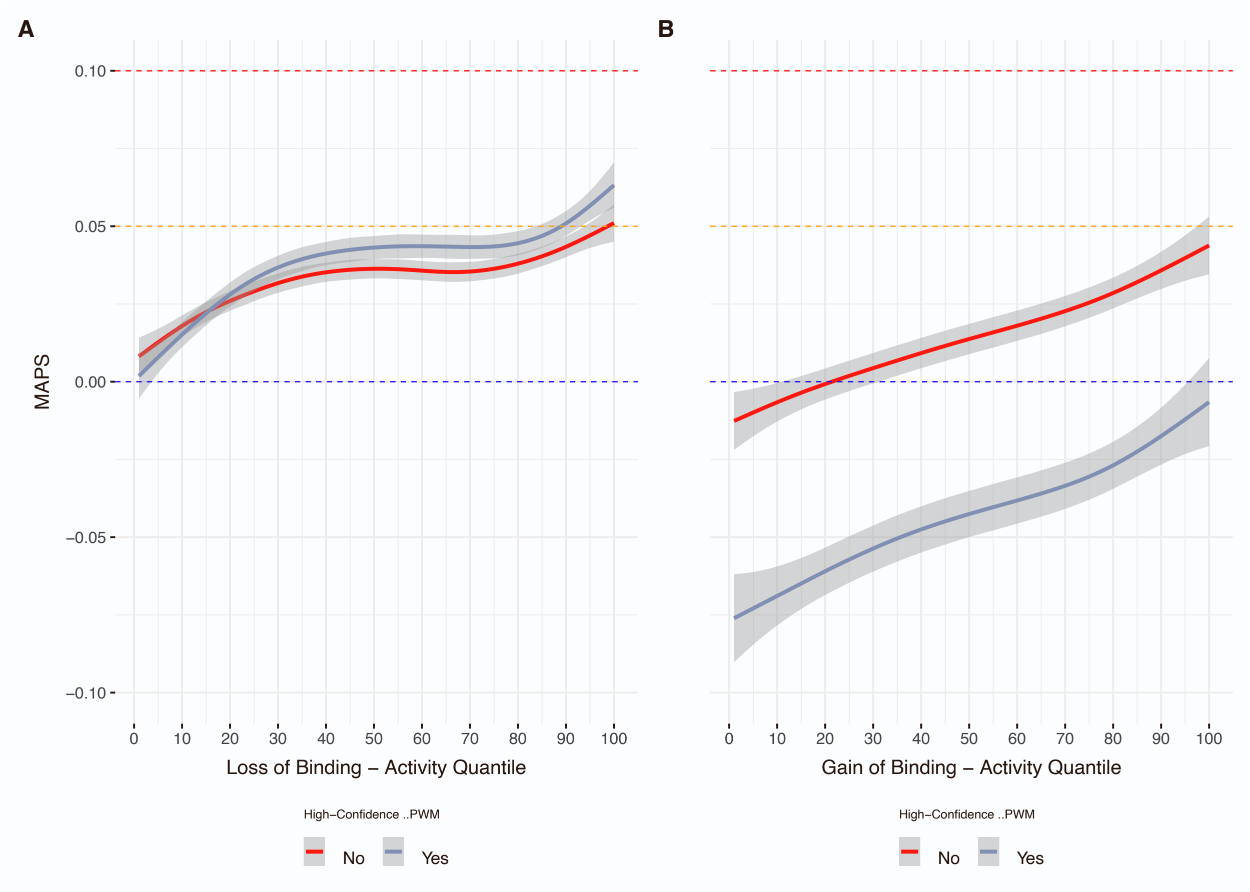


**Figure S6. A)** Relationship between allele frequency as measured by MAPS and the loss of CTCF binding activity stratified by confidence in the ΔPWM calls. For context, we display MAPS scores for synonymous (blue line), missense (orange) and splicing (red) variants. **B)** Relationship between allele frequency as measured by MAPS and the gain of CTCF binding activity stratified by confidence in the ΔPWM calls. A-B) Plotted are smoothed conditional means using the LOESS method.

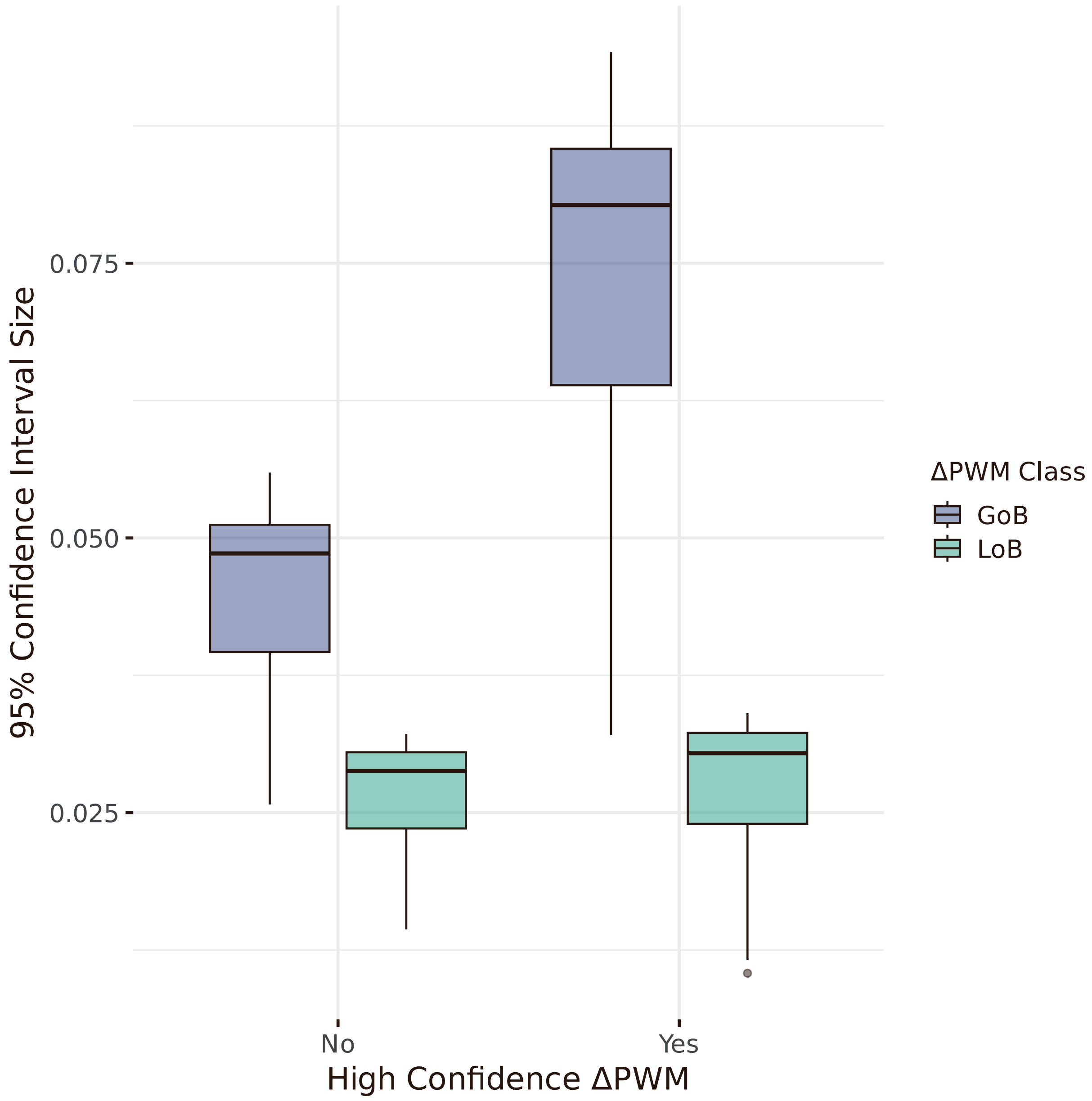


**Figure S7.** Size of 95% confidence intervals on all singleton proportions used to calculate MAPS scores. Confidence was measured by applying a binomial test on the proportion of singletons variants in each binding activity quantile, stratified by the confidence in each variant's ΔPWM score. Gain of binding and loss of binding are abbreviated as GoB and LoB, respectively.

| Annotation | N | Length (bp) | Footprint (Mb) | Coverage Conservation | Coverage CADD | Number SNVs |
|---|---|---|---|---|---|---|
| rDHS | 1063878 | 273 | 290 | -- | -- | -- |
| rDHS + PWM | 355418 | 15 | 5 | 100 | 0.24 | 1253229 |

**Table S1. Summary statistics of the study dataset**. Statistics describing the noncoding sequence footprint of putative CBSs identified through our approach, stratified by whether the CBS has both rDHS and PWM support. The fields are as follows; N is the number of sequence elements in each group, Length is the mean length of all sequences for a given group in basepairs, footprint is the cumulative amount of genomic sequence covered through each annotation approach, coverage conservation describes the proportion of sequence annotated with evolutionary sequence conservation (GERP++, LINSIGHT, PhyloP100 and Phastcons100), Coverage CADD is the proportion of sequence annotated with CADD scores and Number of SNVs refers to the total number of overlapping SNVs in gnomAD. Dashed lines (--) signal data we did not collect as a part of this study.

| Variant Class | N | MAPS | SEM |
|---|---|---|---|
| Synonymous | 2926343 | 0.0000 | 0.0003 |
| 3’ UTR | 10000000 | 0.0275 | 0.0002 |
| Intergenic | 19999998 | 0.0286 | 0.0001 |
| Missense | 6075537 | 0.0435 | 0.0002 |
| 5’ UTR | 9442976 | 0.0558 | 0.0002 |
| Splice Donor | 112806 | 0.0965 | 0.0015 |
| Start Lost | 26198 | 0.0985 | 0.0030 |
| Stop Lost | 12784 | 0.1122 | 0.0043 |
| Stop Gained | 201570 | 0.1196 | 0.0011 |
| Splice Acceptor | 82838 | 0.1256 | 0.0017 |

**Table S2.** MAPS scores for different functional classes of genic variation in gnomAD. Error was calculated as the standard error of the mean for each proportion. Variant class indicates the Variant Effect Predictor (VEP) worst consequence. The count of 3’UTR and Intergenic variants were limited to a random sample of the displayed size to facilitate their processing.
